# Bio‐Inspired, Zwitterionic Copolymers with Amphiphilic Character

**DOI:** 10.1002/marc.202401099

**Published:** 2025-03-27

**Authors:** Theresa M. Lutz, Cevin P. Braksch, Jonas De Breuck, Matthias Hartlieb, Meike N. Leiske

**Affiliations:** ^1^ Macromolecular Chemistry University of Bayreuth Universitätsstraße 30 95447 Bayreuth Germany; ^2^ Institute of Chemistry University of Potsdam Karl‐Liebknecht‐Str. 24–25 14476 Potsdam Germany; ^3^ Fraunhofer Institue for Applied Polymer Research (IAP) Geiselbergstraße 69 14476 Potsdam Germany; ^4^ Bavarian Polymer Institute Universitätsstraße 30 95447 Bayreuth Germany

**Keywords:** arginine, cell association, polyelectrolyte, XPI RAFT polymerization, zwitterionic polymer

## Abstract

Selectively targeting diseases with therapeutics remains a crucial yet still unsatisfied challenge in (nano)medicine. In recent years, a large body of biologically based drug carrier systems are produced which have proven to be suitable for the efficient transport of active compounds such as biopharmaceuticals and biotechnological drugs. However, those naturally occurring materials often entail risks, for example, due to accessible, functional groups created by uncontrolled protein denaturation processes of enzymes (e.g., proteases) which can lead to unwanted side effects in the body. To deal with this issue, designing bio‐inspired synthetic copolymers offers a suitable alternative compared to systems based on materials derived from natural sources. Owing to the variety of electrostatically interacting motifs abundant in nature, synthetic statistical copolymers are developed with different polarity and zwitterionic arginine‐derived units. To achieve the required physicochemical demands, a simple one‐step synthesis approach is applied, the so‐called xanthate‐supported photo‐iniferter reversible‐addition‐fragmentation chain‐transfer (XPI‐RAFT) polymerization. The cellular association of these polymers is compared to a fully non‐ionic polymer. The results highlight new findings in the design of zwitterionic macromolecule structures for medical applications and further progress the understanding of the driving forces of the cell specificity of polyzwitterions.

## Graphical abstract

Zwitterionic copolymers based on arginine‐derived monomers are produced by xanthate‐supported photo‐iniferter reversible‐addition‐fragmentation chain‐transfer polymerization. Experiments in relevant conditions show that the designed (co)polymers hardly interact with biologically relevant proteins and enzymes such as bovine serum albumine (BSA), fetal bovine serum (FBS), and lysozyme, which is modulated by the hydrophobicity based on the comonomer ratio.

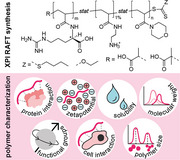

## 1 Introduction

Freely administered drugs often fail to reach the target side and can be easily degraded by endogenous metabolic pathways. To deal with those physiological challenges, scientists have developed carrier systems that protect the compounds and transport/release them on demand.^[^ ^1^ ^]^ Indeed, numerous studies demonstrated the successful delivery of cargo into targeted cells via multiple uptake mechanisms.^[^ ^2^ ^]^ The ability to form protective carriers that can overcome challenging biological conditions (e.g., various enzymes, changing pH levels) is pivotal.^[^ ^3^ ^]^ Carriers based on endogenous molecules,^[^ ^4^ ^]^ for example albumin, preserve the excellent biocompatibility of proteins (i.e., non‐toxic degradation products) and ensure material stability.^[^ ^5^ ^]^ However, bio‐derived materials entail challenges including limited commercial availability,^[^ ^6^ ^]^ in certain cases, the complex production of recombinant (oligo‐)peptides,^[^ ^7^ ^]^ reproducibility,^[^ ^4^ ^]^ and the tendency of surface adsorption (e.g., via various functional groups) such as syringes during application.^[^ ^8^ ^]^ Therefore, (semi‐)synthetic alternatives typically serve as substitutes to reduce costs and tune drug carrier properties. Examples of such engineered drug carriers are dendrimers^[^ ^9^ ^]^ and lipid‐^[^ ^10^ ^]^ or peptide‐modified^[^ ^11^ ^]^ copolymer variants. Those selected molecules and motifs allow for specific interactions^[^ ^12^ ^]^ and/or particle formation.^[^ ^13^ ^]^ One common feature of copolymers is the tailored combination of building blocks required for an individual solution to meet high flexibility for multifaceted applications.^[^ ^14^ ^]^ To represent an ideal material for drug transport and/or specific cell interaction, the charge profile and the hydrophobicity/hydrophilicity have to be well‐controlled.^[^ ^15^ ^]^ The other necessary parameters that should be addressed to obtain an ideal drug carrier for medical applications are stiffness, size, and shape. However, the charge profile is one common tunable feature,^[^ ^16^ ^]^ contributing to successful specificity toward malignant cells.^[^ ^17^ ^]^ In this context, amino acids are interesting building blocks as they play an important role in cancer proliferation as a nutritional source.^[^ ^18^ ^]^ Carrier systems with those motifs may provide excellent reproducibility and the most cost‐efficient option in cancer therapy.^[^ ^19^ ^]^ Zwitterionic amino acids act as target groups for cancer cells,^[^ ^20^ ^]^ since the amino acid transporters are upregulated compared to healthy cells.^[^ ^18^ ^]^ Moreover, they reduce protein adsorption (e.g., in serum)^[^ ^21^ ^]^ and confer pH‐responsive properties to the carrier system which could be very promising for cellular internalization and/or endosomal escape. One recent example investigated the potential of copolymers modified with arginine and histidine.^[^ ^22^ ^]^ The positively charged guanidinium group of arginine enabled cell membrane penetration (= internalization of the carrier system) at physiological pH value and the cationic imidazole group of histidine facilitated endosomal escape of the carrier system at acidic pH conditions.^[^ ^22^ ^]^

Advanced in reversible deactivation radical polymerization (RDRP) techniques such as reversible addition‐fragmentation chain‐transfer (RAFT) polymerization^[^ ^23^ ^]^ have enabled increasingly easy access to highly defined copolymers. In particular, photo‐induced strategies enable precise control and high‐end group fidelities.^[^ ^24^ ^]^ Photo‐iniferter (PI)‐RAFT polymerization is particularly elegant as the chain transfer agent (CTA) used to control the polymerization is also used as a photo‐initiatior.^[^ ^25^ ^]^ To broaden the scope of this technique while maintaining its efficiency, we recently developed xanthate supported (X)PI‐RAFT polymerization where a mixture of CTAs is used.^[^ ^26^ ^]^ In this technique, the combination of a xanthate and a second CTA (here a trithiocarbonate) enables rapid production of polymers while maintaining good control over a broader variety of monomers.^[^ ^27^ ^]^ Produced polymers can also be chain‐extended easily, though this is not in focus in the present study. We have employed this technique for the production of amino acid‐based antimicrobial polymers,^[^ ^28^ ^]^ and the synthesis of polymer‐protein conjugates.^[^ ^27^ ^]^

Herein, we use XPI‐RAFT polymerization of amino acid monomers to produce customized bio‐inspired zwitterionic copolymers. Initially, we investigate how these zwitterionic polymers interact with different proteins (e.g., lysozyme) and cells, and how different pH values affect the material (e.g., change in the charge profile). Those characteristics help to establish a pre‐defined copolymer concept and ‐design for complicated, pathological scenarios in the future.

## 2 Results and Discussion

### 2.1 Synthesis of Arginine Acrylamide (Arg‐OH‐AAm)

**Figure 1:**
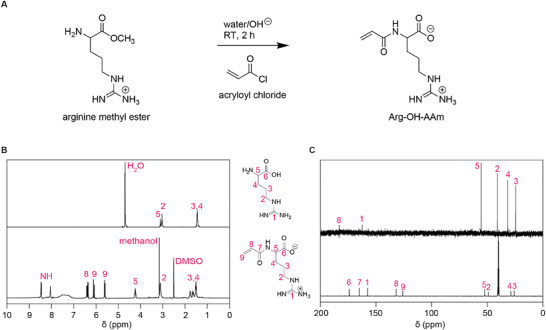
Synthesis scheme and NMR data to show and characterize the monomers investigated in this study. A) Schematic Representation of the monomer synthesis. B) ^1^H NMR spectra (L‐arginine: 300 MHz, D_2_O; Arg‐OH‐AAm: 400 MHz, DMSO‐d6). C) ^13^C NMR spectrum of L‐arginine (75 MHz, D_2_O) and Arg‐OH‐AAm (100 MHz, DMSO‐d6).

To produce the zwitterionic, arginine‐derived polymers, we chose vinyl monomers to polymerize them by RAFT polymerization. In a first step an arginine‐based acrylamide monomer was synthesized (**Figure** **1**A). Due to the limited solubility of amino acids in organic media, L‐arginine methyl ester dihydrochloride was chosen as the starting material. The α‐carbon amino group served as a reactive handle for the acrylamide formation via the reaction with acryloyl chloride. To demonstrate the successful formation of arginine acrylamide (Arg‐OH‐AAm), both L‐arginine and Arg‐OH‐AAm were analyzed by proton nuclear magnetic resonance (^1^H NMR) spectroscopy (Figure 1B). The success of the amide formation was confirmed by the downfield shift of the signal corresponding to the CH‐group adjacent to the newly formed bond from *δ* = 3.1 ppm (L‐arginine) to *δ* = 4.2 ppm (Arg‐OH‐AAm). In addition, the monomeric vinyl signals of Arg‐OH‐AAm (*δ* = 5.5 to 6.5 ppm), as well as the protons of the amide bond (*δ* = 8.0 ppm) suggested the formation of the desired product. The visibility of the guanidinium protons (*δ* = 8.5 ppm) further indicated the absence of a by‐product in which the guanidinium group reacted with acryloyl chloride instead. Notably, during the reaction the methoxy group was hydrolyzed to a carboxy group, visible by the absence of the characteristic methyl ester peak at *δ* = 3.7 ppm.^[^ ^22^ ^]^ Thus, we could produce the desired monomer in a one‐step process, rendering a deprotection step unnecessary. To further confirm purity, carbon nuclear magnetic resonance (^13^C NMR) spectroscopy was applied (Figure 1C). In particular, the chemical shift of the CH‐group adjacent to the α‐amino acid group (δ = 52.4 ppm) confirmed product formation via the reaction of the α‐amino group. Furthermore, the signal of the quarternary carbon of the guanidinium group (δ = ≈157.6 ppm) confirmed the absence of an undesired guanidin functionalization. Next, we aimed to polymerize the arginine‐derived vinylic zwitterionic monomer Arg‐OH‐AAm directly.

### 2.2 Synthesis of Statistical Copolymers with Arginine Motifs

**Scheme 1:**
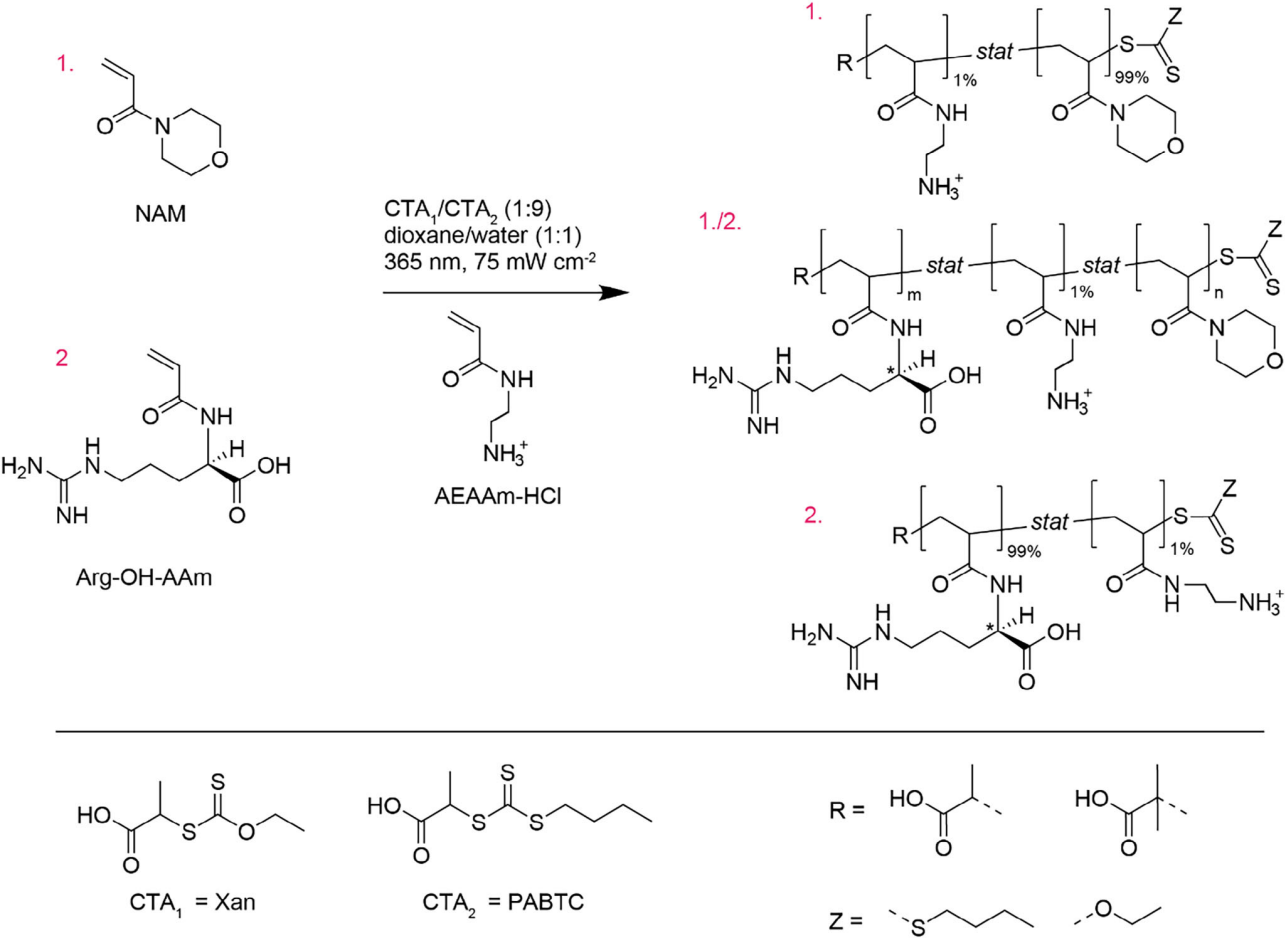
Schematic representation of the polymer synthesis strategy. XPI RAFT polymerization of statistical copolymers is based on the conversion of monomers (NAM, AArg‐OH‐AAm, and AEAAm‐HCl) with the CTAs Xan and PABTC (ratio of 1:9) in dioxane/water mixtures (1:1) using UV irradiation (λ = 365 nm, 75 mW cm^−2^).

It has been described previously that vinylic monomers were suitable for synthesizing amino‐acid‐derived functional (co)polymers by RDRP techniques, e.g. via RAFT polymerization.^[^ ^15^, ^17^, ^29^ ^]^ While CTAs are sensitive to aminolysis and usage of unprotected primary amino groups should be limited, the polymerization of an arginine‐derived acrylamide with an unprotected guanidine moiety has been shown before.^[^ ^22^, ^30^ ^]^ Here, we wanted to extend this potential to the fully unprotected Arg‐OH‐AAm monomer applying XPI‐RAFT polymerization.^[^ ^26^ ^]^ Furthermore, to enhance the solubility of the resulting zwitterionic polymers, it was aimed at synthesizing biocompatible copolymers comprising non‐ionic *N*‐acryloylmorpholine (NAM) moieties.^[^ ^31^ ^]^ The synthetic strategy was based on a one‐step reaction (**Scheme** **1**). For this purpose, the monomers NAM, and Arg‐OH‐AAm were co‐polymerized in the presence of two (CTAs), namely 2‐[(Ethoxythioxomethyl)thio]‐2‐methylpropanoic acid (Xan) and propanoic acid butyl trithiocarbonate (PABTC), via XPI‐RAFT. The polymerization was performed using high‐intensity ultraviolet (UV) light at 365 nm to obtain P(Arg‐OH‐AAm‐*stat*‐NAM) copolymers with varying monomer ratios, adjusted to achieve the required arginine content of 0, 25, 50, 75, and 100% per copolymer (Figure S1 and Tables S1 and S2, Supporting Information). The primary amine‐containing monomer aminoethylacrylamide hydrochloride (AEAAm‐HCl) was further incorporated at a 1% feed ratio to facilitate fluorescent labeling at a later stage. For reasons of clarity, the AEAAm‐HCl moieties are not referred to in the polymer names (P(Arg‐OH‐AAm), P(Arg‐OH‐AAm_n%_‐*stat*‐NAM_m%_).

**Figure 2:**
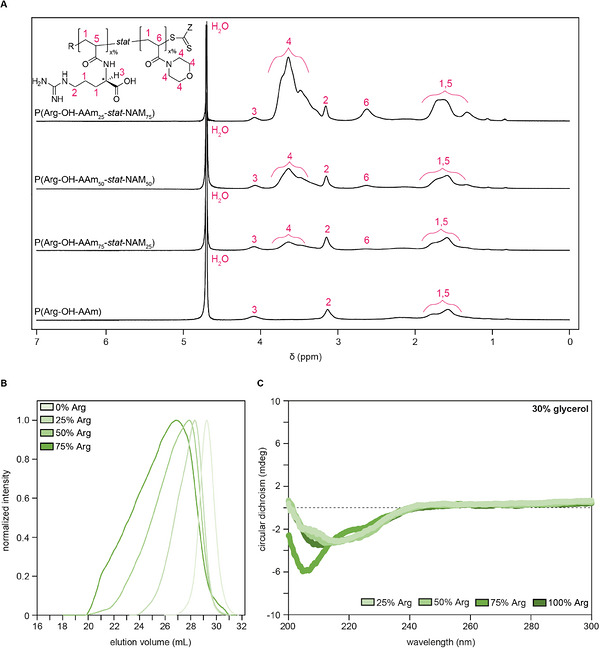
Characterization of the synthesized copolymers. A) ^1^H NMR spectra (300 MHz, H_2_O) of purified (co)polymers and Arg‐OH‐AAm. Spectra were normalized to the signal at *δ* = 4.1 ppm. B) SEC in 80:20 Water/acetonitrile (ACN) mixture containing 0.1 M NaCl, and 0.1 V% trifluoroacetic acid (TFA) (poly(2‐vinylpyridine) (P2VP)‐cal.). C) Representative circular dichroism curves showing the stereochemistry of P(Arg‐OH‐AAm_n%_‐*stat*‐NAM_m%_) and P(Arg‐OH‐AAm).

The formation of the copolymers was followed by ^1^H NMR spectroscopy of the reaction mixtures and the purified compounds (Figure 1A; Figure S2, Supporting Information). Indeed, the conversion of the typical acrylic resonance signals (*δ* = 5.8–6.5 ppm) to the aliphatic backbone signals (*δ* = 1.5–1.8 ppm) was observed. The complete disappearance of the acrylic signals within 120 min of reaction time (Figure S2, Supporting Information) indicated quantitative monomer conversion for all (co)polymers (Table S3, Supporting Information). The methylene signals in the side‐chain of the Arg‐OH‐AAm units and the proton resonance signal of α‐carbon were detectable at *δ* = 3.2 ppm (L‐Arg: *δ* = 3.0 ppm) and *δ* = 4.2 ppm (L‐Arg: *δ* = 3.2 ppm), respectively, for all copolymers. Copolymers with NAM further contained the characteristic resonance signals of the morpholine ring at *δ* = 3.3–3.6 ppm (**Figure** **2**A).

To gain further insights into the (co)polymer properties, we investigated the molar mass distribution of each (co)polymer by aqueous size exclusion chromatography (SEC). While the polymer without incorporation of Arg‐OH‐AAm showed the expected narrow molar mass distribution, increasing percentages of the functional comonomer broadened the distribution markedly (Figure 2B). This could be ascribed to a loss of control during the polymerization of such a challenging comonomer, however, the reason could also be sought in column interaction by the zwitterionic moieties under the acidic buffer conditions of the SEC eluent. Still molar masses are in the expected range (Table S3, Supporting Information). Similar trends were obtained when the eluent was changed to H_2_O supplemented with NaCl/formic acid (Figure S3, Supporting Information).

Having successfully demonstrated that arginine‐based monomers can be integrated into polymers, we were interested in their bioactivity. Indeed, L‐arginines hold the potential to be used to positively affect biological processes. For example, L‐arginines were shown to combat neurotoxicity^[^ ^32^ ^]^ and initial experiments proved their benefit for cancer therapy.^[^ ^33^ ^]^ Therefore, further copolymer characterizations provide information on the structural and physicochemical properties in the following section.

### 2.3 Characterization of Arginine‐Derived, Zwitterionic Copolymers

In preliminary solvation experiments (data not shown), it was found that for polymers with high Arg‐OH‐AAm contents, the solubility in aqueous media/buffer was reduced. To use the polymers for further experiments in a physiological environment, a solvent had to be found that is not toxic (e.g., for cells). Indeed, the copolymer with 100% Arg‐OH‐AAm content was insoluble in, e.g., phosphate buffer (Figure S4A, Supporting Information), however, the addition of 30% glycerol allowed for efficient solubilization. One important analysis of the copolymers was to identify the stereochemistry of the polymers since enantiomeric configurations have played a crucial role in the efficacy of bioactive compounds.^[^ ^34^ ^]^ The negative signals obtained by circular dichroism (CD) measurements for each of the different copolymers in glycerol/Dulbecco's phosphate buffered saline (D‐PBS) as solvent (Figure 2C) indicated that the majority of arginines in the polymers exist as L‐amino acid, verifying the stability of the stereocenter during monomer synthesis and polymerization.^[^ ^35^ ^]^ Measurements in pure D‐PBS verified these results (Figure S1F, Supporting Information).

**Figure 3:**
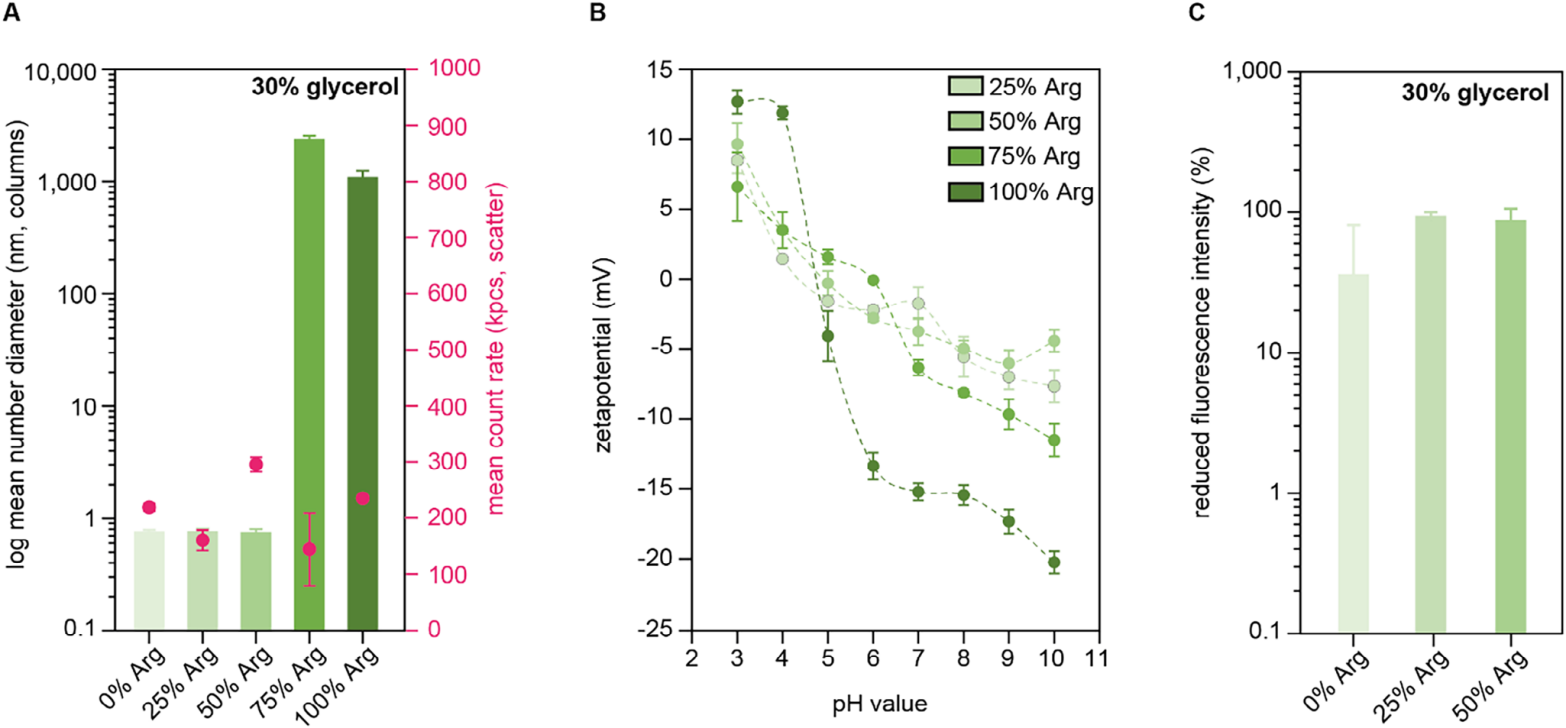
Assessment of copolymer polarity and polymer characterization in solutions with different pH values. A) The mean number diameter and the mean count rates of the formed copolymers with various Arg‐OH‐AAm motif amounts used in this study are determined by DLS. B) To evaluate the isolelectric point (pI) of each copolymer, zeta potential measurements are investigated at pH values in the range of 3 and 10. The dashed lines do not show measured data points. C) The reduced fluorescent intensity in the aqueous phase after treatment with an organic solvent in a phase separation setup indicates how the Arg‐OH‐AAm content influences the polarity. All error bars denote the standard error of the mean as obtained from n = 3 independent analytical triplicates.

The hydrodynamic diameter of copolymers was studied by dynamic light scattering (DLS) measurements. Since the copolymer with 100% arginine content was insoluble in D‐PBS (Figure S4A, Supporting Information), the addition of 30% glycerol allowed for efficient solubilization and measurement of all polymers. The mean number diameter of the copolymers was ≈1 nm (≈fivefold to ≈eightfold smaller than in D‐PBS) for Arg‐OH‐AAm contents up to 50% per copolymer (higher non‐ionic NAM content; **Figure** **3**A) indicating single‐chain coils and the absence of major aggregates. In comparison, copolymers with higher zwitterionic motif ratios (i.e., 75% and 100% Arg‐OH‐AAm) showed diameters of ≈3000 nm (copolymer in glycerol/D‐PBS: 75% Arg‐OH‐AAm content) and ≈1000 nm (copolymer in D‐PBS: 75% Arg‐OH‐AAm content; copolymer in glycerol/D‐PBS: 100% Arg‐OH‐AAm content; Figure 3A). The formation of these larger aggregates is potentially attributed to the increased hydrophobicity in the zwitterionic state^[^ ^29^ ^]^ and may occur at physiological pH conditions. In conclusion, the hydrodynamic size measurements indicate that polymers with high arginine contents (≥ 75%) can form intermolecular interactions.

Given the fact that polyelectrolytes, and in particular amino‐acid‐derived polyzwitterions, are highly pH‐responsive,^[^ ^29^ ^]^ we further studied their response to changes in pH value by means of electrophoretic light‐scattering (ELS) measurements. We have calculated the pI of the copolymers to be 4.47 (25% Arg‐OH‐Aam), 4.93 (50% Arg‐OH‐Aam), 5.99 (75% Arg‐OH‐AAm), and 4.75 (100% Arg‐OH‐AAm) from the ζ‐potential measurements. Consequently, the zwitterionic character of Arg‐OH‐AAm at neutral pH values (p*K*_a_ value of arginine guanidinium group: 13.8^[^ ^36^ ^]^) and the high content of Arg‐OH‐AAm within polymers comprising 75% or 100% Arg‐OH‐AAm units have an important impact on the agglomeration in glycerol/D‐PBS since the high density of charged groups facilitate intermolecular interactions (ζ‐potential between ‐15 mV and ‐5 mV, Figure 3B). In other words, since both, α‐carboxyl and the guanidinium units are charged,^[^ ^37^ ^]^ intermolecular electrostatic interaction led to the formation of agglomerates. Moreover, those copolymer solutions became turbid (data not shown) which indicated aggregation (Figure S4B, Supporting Information).^[^ ^29^ ^]^ This hydrophobicity not only affects the copolymer insolubility; those molecules are also less prone to electrostatic interactions resulting in a lower zeta potential compared to the copolymers with lower arginine content.^[^ ^38^ ^]^ Conversely, no turbidity of the solution was observed (Figure 3B) for the copolymers with 25% and 50% Arg‐OH‐AAm content, since the amount of Arg‐OH‐AAm motifs is lower and less electrostatic interactions occurred. This was also indicated by the slightly negative ζ‐potential of these copolymers at elevated pH values. In addition, the presence of NAM as a pH‐independently hydrophilic building block further facilitated the solubilization.

Hydrophobicity of zwitterionic (co)polymers is an important parameter, which impacts cell specificity.^[^ ^29^ ^]^ Therefore, we analyzed the impact of comonomer ratios at the pH value of the cell culture medium (pH 7.4), since this can provide information on the cell membrane interaction. For this reason, polymers were fluorescently labeled via their AEAAm‐group with Cy5‐*N*‐hydroxysuccinimide (NHS) ester (labeling process shown in Scheme S1, Supporting Information; SEC measurements and corresponding M_n_ values, see Table S4 and Figure S8A, Supporting Information), and the hydrophilicity was studied by determination of polarity ratios in a glycerol/D‐PBS and chloroform mixture as previously reported.^[^ ^39^ ^]^ We observed that the fluorescent labeling further decreased the polymer solubility in gylcerol/D‐PBS, especially the copolymers with 75% and 100% Arg‐OH‐AAm content (Figure S8B, Supporting Information). Therefore, we only investigated the (co)polymer variants with 0%, 25%, and 50% Arg‐OH‐AAm content. The quantification of the reduced fluorescence intensity in the water phase allowed for the determination of the polymer hydrophobicity. Based on this data, as expected, the experimental results obtained revealed that the hydrophilicity was slightly reduced with increasing Arg‐OH‐AAm amount (Figure 3C). This, in turn, means that Arg‐OH‐Aam, despite its charged character, possesses hydrophobic qualities, explaining the aggregates observed via DLS measurements.

**Figure 4:**
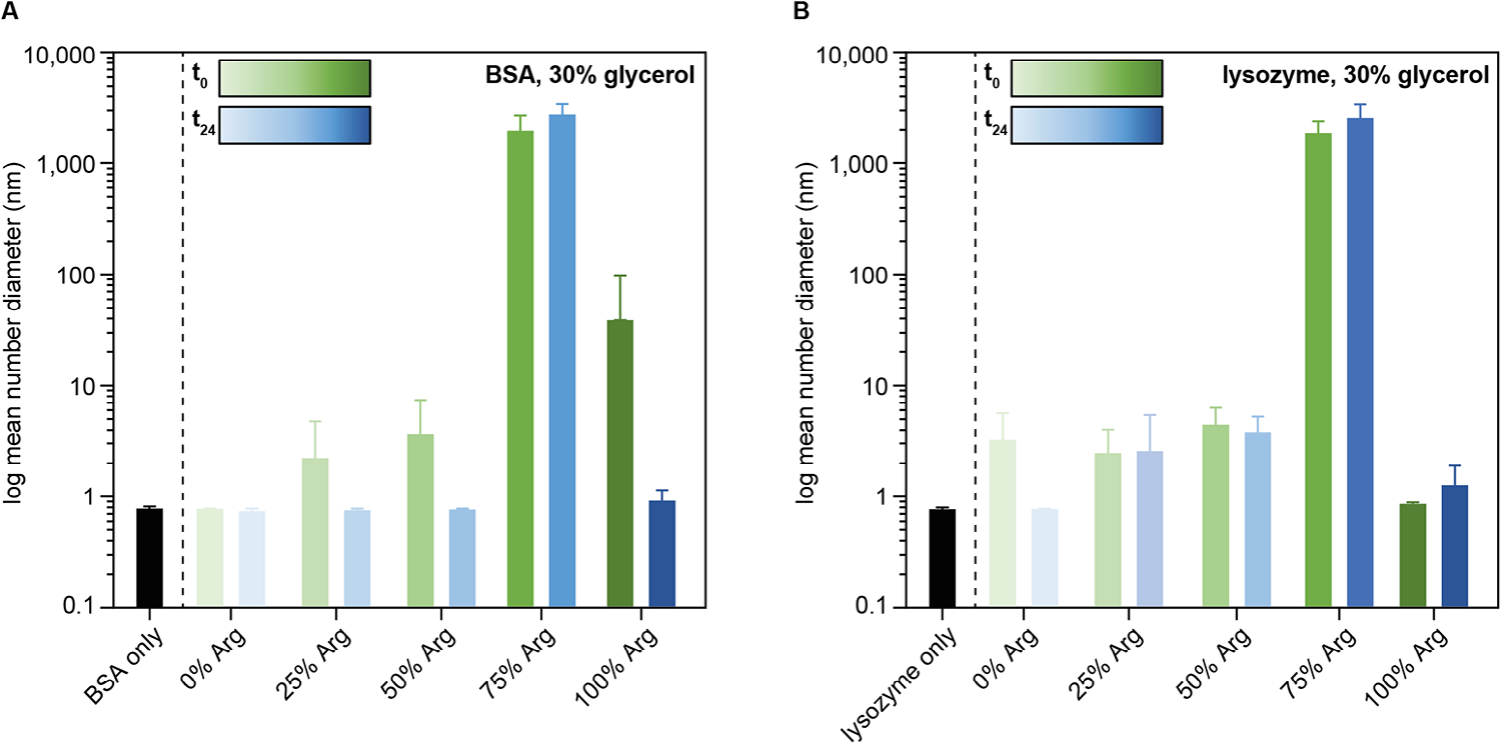
Copolymer interaction with proteins. A,B) Unspecific binding interactions with proteins (BSA and lysozyme only, black) are assessed immediately after mixing (t_0_) and after 24 h incubation time at 37 °C. All error bars denote the standard error of the mean as obtained from n = 3 independent analytical triplicates.

During application, copolymer‐biomolecule interactions could affect, e.g., polymer aggregation, charge profile, and cellular uptake. A suitable carrier system should resist unspecific binding interactions to maintain its integrity. Therefore, we have evaluated the putative interaction with two representative proteins of opposite charge^[^ ^40^ ^]^ namely bovine serum albumin (BSA, negative net charge^[^ ^41^ ^]^; pI between 4.6 and 5.1^[^ ^42^ ^]^) and lysozyme (positive net charge^[^ ^43^ ^]^; pI of ≈11.0^[^ ^44^ ^]^). Results from the literature have reported a compaction of native proteins^[^ ^45^ ^]^ and a stability increase of BSA and lysozyme when those molecules are dissolved in glycerol‐containing solutions.^[^ ^46^ ^]^ This finding supports our data, since BSA and lysozyme exhibit a mean number diameter of ≈6 nm and ≈4 nm, respectively, while in D‐PBS with 30% glycerol a diameter of ≈0.8 nm (both proteins) was observed (Figure S4C, Supporting Information). However, since glycerol has an influence on the solvent viscosity, the diffusion rate of the polymers was affected during DLS measurements. Thus, the decrease in the mean number diameter was only qualitative. Most tested copolymers showed limited protein interactions in glycerol at physiological pH conditions, independent of the charge state of BSA and lysozyme, respectively (Figure S5, Supporting Information (size distribution), Figure S6, Supporting Information (number distribution), **Figures** **4**A,B, and S7, Supporting Information (polymers only)). The mean number diameters at the beginning of the measurements (t_0_) were very similar to values obtained after 24 h (t_24_) (Figure 4; Figure S4D,E, Supporting Information). Only the polymer with 75% Arg‐OH‐AAm content exhibited mean number diameters of ≈3000 nm, which was comparable to the measurements without proteins (Figure 3A), making a conclusion toward protein‐polymer interactions difficult. However, when comparing the size distribution of the copolymers (Figure S7, Supporting Information) and copolymers interacting with BSA or lysozyme (Figure S5, Supporting Information), the results agreed with this picture of copolymer/protein interaction. In detail, the peaks located at ≈1 nm and ≈20 nm size diameter correspond to BSA/lysozyme signals and were shifted to larger size diameters after the addition of copolymers (75% Arg‐OH‐AAm content; Figures S5 and S7, Supporting Information).

The mean number diameters obtained from copolymers incubated in cell medium were very similar to those obtained for cell medium with fetal bovine serum (FBS; Figure S4F, Supporting Information). Accordingly, those copolymers could be engaged in cell interaction experiments, as interactions with proteins in cell medium (e.g., FBS) were negligible.

In summary, we conclude that the synthesized and non‐fluorescently labeled (co)polymers are soluble in glycerol/D‐PBS. Protein aggregation and unspecific protein‐polymer interaction are mainly governed by high arginine contents within the copolymer (75% and higher), which is also attributed to the hydrophobic character of arginine monomers.

### 2.4 Interaction Capability of Zwitterionic Copolymers with Eukaryotic Cells

**Figure 5:**
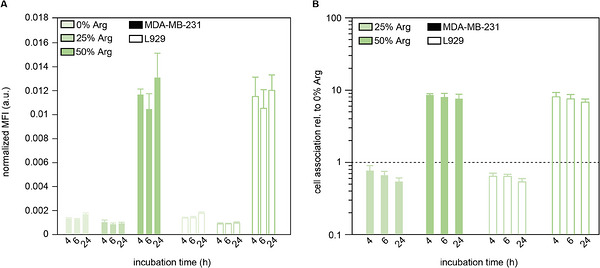
Interaction of copolymers with eukaryotic cells. A) The quantification of Cy5‐labeled copolymers interacting with cancer and non‐cancer cells was assessed by flow cytometer measurements. (B) The cell association data show how the interaction with fluorescent polymers changes over time compared to the negative control (polymer with 0% Arg‐OH‐AAm). B,C) All error bars denote the standard error of the mean as obtained from n ≥ 3 samples.

We next investigated the polymer interactions with eukaryotic cells. We initially assessed the metabolic activity of the different copolymers with L929 mouse fibroblasts as per ISO 10993–5 standard.^[^ ^47^ ^]^ The results revealed that no inhibitory cytotoxic effects were observed at concentrations ≤ 0.16 mg mL^−1^. However, we found reduced (< 70%) cell viability for some copolymers (e.g., copolymers with 50% and 0% Arg‐OH‐AAm motifs) at concentrations ≥ 0.63 mg mL^−1^ (Figure S9, Supporting Information). Comonomer hydration modulated by high NAM contents might affect the solubility of those (co)polymers and cause cytotoxic effects at higher concentrations.^[^ ^17^ ^]^ When using a copolymer concentration of 0.04 mg mL^−1^, we found excellent metabolic activity, and the fluorescence intensity of Cy5‐labeled polymers (necessary for the detection of cell interacting polymers) was expected to be sufficient as well (Figure S8B, Supporting Information). Given the insolubility of Cy5‐labeled copolymers with 75% and 100% Arg‐OH‐AAm content in aqueous environments (Figure S8B, Supporting Information), they were not considered for interaction and cell uptake studies (gating strategy for flow cytometry measurements, Figure S10, Supporting Information) with MDA‐MB‐231 cancer and L929 non‐cancer cells. It has been described previously that the NAM homopolymer interacts non‐specifically with both cell types, cancer cells and non‐cancer cells.^[^ ^17^ ^]^ This limited cell specificity agreed well with the results obtained (independent of the incubation time) and thus, copolymers with 0% Arg‐OH‐AAm were the control samples (**Figure** **5**A). We found a reduced cell association of 25% Arg‐OH‐AAM polymers compared to NAM controls for both cell lines (up to 50% reduced) over time. This might be explainable by the hydrophilicity and/or the abundance of zwitterionic motifs contained in the polymer (25% Arg‐OH‐AAM content) since interactions with hydrophobic polymers are favored^[^ ^29^ ^]^ as well as charge profiles influence interactions with biological barriers.^[^ ^48^ ^]^ The prevailing hydrophilicity of the copolymer backbone and even the presence of 25% Arg‐OH‐AAm motifs was insufficient to achieve cell specifity (results similar for both cell lines at all time points). Once the copolymeric Arg‐OH‐AAm content is increased to 50% and thus the hydrophobicity (caused by the zwitterionic motifs) of the copolymers, the binding affinity to both cell types was ≈sixfold higher compared to the pure PNAM control (but no cell specificity occurred; Figure 5A). By means of hydrophobicity, the polymers were able to interact, especially with the lipid bilayer membrane of cells.^[^ ^29^ ^]^ Over time, the amount of bound Arg‐OH‐AAm motifs and the hydrophobicity of the copolymer backbone system (50% Arg‐OH‐AAm content) were sufficient to interact ≈sevenfold to ≈eightfold higher with cells than the negative control, whereas the hydrophilic ones (25% Arg‐OH‐AAm motifs) showed lower cell association compared to NAM (Figure 5B).

## 3 Conclusion

Here, we synthesized a novel arginine acrylamide monomer which served as a building block for the formation of zwitterionic copolymers with varying polarity by XPI‐RAFT polymerization. After calculation of the molar mass and successful monomer conversion of arginine acrylamides and copolymer polymerization by NMR and SEC measurements, respectively, the chirality of the amino acid functionalization was observed by circular dichroism. The majority of arginines in L‐conformation entail copolymers with potential application for pathological scenarios such as cancer and neurological diseases. However, we expect that alterations in the copolymer composition might affect the properties. The obtained (co)polymers can modulate polymer aggregation by the rising availability of hydrophobic and zwitterionic arginine motifs, which influences the solubility of the formed polymers. This implies that the presence of multiple hydrophobic groups may limit the biological application in aqueous environments. However, experiments in relevant conditions show that the designed (co)polymers hardly react with biologically relevant proteins and enzymes such as BSA, FBS, and lysozyme. This feature is important when those copolymer systems are applied to the body in the future since possible bio‐fouling interactions with proteins in the bloodstream are negligible and fast biodegradation by enzymes may be limited. The increasing hydrophobicity, caused by the amount of arginine motifs, can modulate high‐affinity interactions with cells. This implies that the application of those (co)polymers in combination with drugs may also support the transport of bioactive compounds to cells and organs. In addition to implementing zwitterionic motifs for cellular interactions, other functional groups could be incorporated in the copolymer design and improve the material properties such as solubility and/or allow for the release of (pro)drugs. The experimental results indicate the importance of a smart copolymer design and the application of this simple polymerization strategy.

## 4 Experimental Section

### Materials

NAM was obtained from TCI and before polymerization reactions, the inhibitor was removed via an aluminiumoxide flash column. Arginine methyl ester was purchased from Carbolution and used as is. D_2_O and DMSO‐d_6_ for ^1^H NMR analysis were purchased from Deutero. Xan^[^ ^49^ ^]^ and PABTC^[^ ^50^ ^]^ were synthesized according to the literature procedure. The solvents and chemicals required for further analytical methods, characterization studies, and cell experiments were obtained from the following manufacturers: Glycerol (Sigma Aldrich, St. Louis, USA), Dulbecco's Phosphate Buffered Saline (D‐PBS; VWR, Radnor, USA), sodium hydroxide (NaOH; VWR), hydrochloric acid (HCl; VWR), Cyanin5 NHS ester (Cy5; Lumiprobe, Hannover, Germany), dimethylsulfoxid (DMSO; Acros organics, Pittsburgh, USA), Chloroform (Fisher Scientific, Pittsburgh, USA), acetonitrile (ACN, Fisher Scientific), Trifluoroacetic acid (TFA; VWR), Bovine serum albumin (BSA; Carl Roth, Karlsruhe, Germany), lysozyme (Carl Roth), Dulbecco's Modified Eagle Medium (DMEM) Low Glucose (VWR), DMEM/Nutrient Mixture F12 (DMEM/F12; VWR), Fetal Bovine Serum Advanced (BSA; Capricorn Scientific, Ebsdorfergrund, Germany), Penicillin/Streptomycin (Pen/Strep; Capricorn Scientific), Trypsin/EDTA (Capricorn Scientific), thiazolyl blue tetrazolium bromid (MTT; TCl chemicals, Tokyo, Japan).

### Nuclear Magnetic Resonance (NMR) Spectroscopy

The copolymers were dissolved in deuterated water (D_2_O) or DMSO‐d_6_ and measured with a ^1^H NMR spectra (400 MHz) at room temperature. The conversion in % is calculated by the integral of the reaction solvent peak (1 ppm) and the vinyl protons of each monomer (Arg‐OH‐AAm and NAM) at t_0_ and t_120_ according to Equation (1) (data analysis software: SpinWorks, version 4.2.12). Here, A_reaction solvent_ denotes the area under the curve (AUC) of the reaction solvent peak and A_Arg‐OH‐AAm or NAM_ describes the AUC of the monomeric vinyl protons of Arg‐OH‐AAm or NAM.

(1) $$conversion=1-\left(\frac{A_{\mathrm{Arg}-\mathrm{OH}-\mathrm{AAm}\;or\;NAM\;t_{0}}}{A_{reaction\;solvent\;t_{0}}}-\frac{A_{\mathrm{Arg}-\mathrm{OH}-\mathrm{AAm}\;or\;NAM\;t_{120}}}{A_{reaction\;solvent\;t_{120}}}\right)$$

Furthermore, the average degree of polymerization (X_n_) and theoretical mean molar mass (M_n,theo_) were determined by Equations (2) and (3). Here, 200 units correspond to the monomer concentration applied and the resulting average chain length of the individual copolymers. Moreover, M denotes the molar mass of Arg‐OH‐AAm (M = 228.25 g mol^−1^) or NAM (M = 141.17 g mol^−1^).

(2) $$X_{monomer}=200*\frac{conversion_{\arg-\mathrm{OH}-\mathrm{AAm}or\;NAM}}{100}$$

(3) $$M_{n,\;theo\;monomer}=DP_{\arg-\mathrm{OH}-\mathrm{AAm}or\;NAM}\;*M_{\arg-\mathrm{OH}-\mathrm{AAm}or\;NAM}$$

Monomer refers to Arg‐OH‐AAm or NAM. Detailed information for each copolymer is listed in Table S3 (Supporting Information).

### Size‐Exclusion Chromatography (SEC) Analysis

The mean molar mass of the different copolymers whether or not fluorescently labeled was conducted on an instrument consisting of a column set with a NOVEMA Max precolumn (particle size = 10 µm) and three NOVEMA Max main columns (particle size = 10 µm, 1 × 100 Å; 2 × 3000 Å) with separation range from 100 to 3 000 000 Da (PSS, Mainz, Germany) together with a variable wavelength detector (1200 Series, Agilent Technologies). As solvent 80:20 Water/ACN mixture + 0.1 M NaCl + 0.3 V% TFA was used (for dissolving polymer and as eluting solvent) with a flow rate of 0.8 mL min^−1^ and the columns were maintained at room temperature. As internal standard ethylene glycol (HPLC grade) was used. The calibration was done with narrowly distributed Poly(2‐vinylpyridine) (narrowly distributed P2VP homo‐polymers, PSS calibration kit). An injection volume of 40 µL was used for the measurements. The samples were dissolved with a concentration of 2 mg mL^−1^ and filtered through a 0.22 µm PTFE Nylon filter before analysis. The UV‐detector was set to λ = 600 nm for measurements of Cy5‐labeled polymers.

### Synthesis of Arg‐OH‐AAm

To a stirred solution of 20 mmol arginine methyl ester (5.22 g) in water (40 mL), 50 mmol of sodium hydroxide pellets (3.20 g, 2.5 eq.) were added while cooling with an ice bath. This corresponded approximately to a 2.1 N aqueous sodium hydroxide solution. With further ice bath cooling, a solution of 60 mmol acryloyl chloride (90.51 g mol^−1^ 4.89 mL, 3 eq.) in 10 mL acetone was added over 8 min to the aqueous amino ester solution. After the complete addition of the acid chloride, the mixture was allowed to warm up to room temperature and was then stirred for 2 h. Subsequently, the pH of the reaction mixture was reduced with concentrated hydrochloric acid (37%, approx. 8 mL) to ≈2.

The aqueous solution was then extracted with ethyl acetate (3×50 mL) to remove unreacted acryloyl chloride. The water was removed by freeze‐drying. Due to salt formation, it was necessary to dilute the obtained pale‐yellow oil with methanol and repeat the freeze‐drying step at least 4 times to completely remove the water. The product was then dried overnight in a high vacuum to obtain a pale yellow, hygroscopic solid. Acryloyl arginine (AArg‐OH, 228.25 g mol^−1^, 1.97 g) was obtained with a yield of 43%.

### Synthesis of AEAM‐Cl

50.6 mL ethylene diamine (1eq, 0.76 mol, 45.5 g) and 100 mL water were mixed in a 500 mL three‐neck round bottom flask with a condenser and a dropping funnel. Then 37.8 mL concentrated hydrochloric acid (0.6 eq, 0.45 mol) was slowly added while cooling in an ice bath to adjust the pH to 10. After that 160 mL methanol was added to the reaction mixture and stirred for 1 h, at room temperature. After cooling to −20 °C, 43,3 mL of acryloyl chloride (0.7eq, 0.53 mol, 48.0 g) was added slowly. The mixture was stirred for an hour in which a white solid precipitated. The reaction mixture was stored overnight at −20 °C and subsequently, the solvents were evaporated. To purify the product, the white solid mass was separated from the solution and washed three times with isopropanol. The isopropanol solution was cooled to 4 °C to obtain white crystals, which were recrystallized in isopropanol. The monomer was obtained with a yield of 22%.

### Copolymer Synthesis

For polymerization NAM, Arg‐OH‐Aam, and AEAM‐Cl were mixed in different ratios and dissolved in a mixture of dioxane/water (1:1) to reach a total monomer concentration of 1 mol L^−1^ (Table S1, Supporting Information). The reaction vessel was a glass reaction vial (5 mL, Pyrex) closed with a rubber septum and without a stirrer. Xan and PABTC were added in a 1:9 ratio with a total concentration of CTA of 0.01 mol L^−1^, leading to an overall X_n_ of 100 for each polymer (Table S2, Supporting Information). After degassing via nitrogen purging (10 min) the sample was subjected to irradiation under UV light (365 nm, 75 mW cm^−2^) using a Photocube from Thales Nano. During polymerization, the reaction was cooled to maintain a temperature of ≈25 °C. After a complete reaction, the solvents were removed via freeze‐drying.

### Circular Dichroism (CD) Measurements

To investigate the copolymeric amino acid chirality, 0.1 mg mL^−1^ of each Arg‐OH‐AAm containing copolymer was dissolved in D‐PBS or 30% glycerol in D‐PBS and measured in a 1 mm quartz cell using a Jasco J‐815 CD Spectrometer (JASCO Labor‐ and Datentechnik GmbH, Groß‐Umstadt, Germany) at 20 °C. The following scanning conditions were applied to obtain circular dichroism (CD) spectra: 50 nm min^−1^ scanning rate, 1 nm bandwidth, 0.1 nm data pitch, and 8 s data integration time. All measurements (3 accumulations per copolymer) were conducted in a wavelength range of 200–300 nm with solvent background correction.

### Fluorescence Labeling of Copolymers

The labeling process was applied to all copolymer variants (an exemplary labeling scheme for copolymers with ≈100% Arg‐OH‐AAm is shown in Scheme S1, Supporting Information) with the red fluorescent dye Cy5 (NHS‐modified; Lumiprobe GmbH, Hannover, Germany). Here, copolymer modification (0% Arg‐OH‐AAm) is described exemplarily (for more details, see **Table** **1**). First, 50 mg copolymer (2.4×10^6^ mol, 1.0 equiv) and 1.9 mg Cy5‐NHS (2.8×10^6^ mol, 1.2 equiv.) were dissolved in 10 mL DMSO:ddH_2_O (1:1 ratio) and incubated in the dark at room temperature (RT) for 24 h during shaking (100 rpm). To remove unreacted Cy5, the reaction product was dialyzed (MWCO = 3.5 kDa, regenerated cellulose) against ultrapure water for six weeks with daily water exchange. Afterward, the polymer suspension was lyophilized, and the dye functionalization was assessed by SEC (data see Figure S8A, Supporting Information).

**Table 1:** The table lists the amounts, molar mass, material quantity, and equivalent of fluorescent dye (Cy5) and copolymers used for each labeling process.

Polymer/dye	M [kg mol^−1^]	m [mg]	n [mol]	equivalents
P(NAM)/Cy5‐NHS	21.18/0.67	50/1.9	2.4 × 10^−3^/2.8 × 10^−3^	1.0/1.2
P(Arg‐OH‐AAm_25_‐stat‐NAM_75_)/Cy5‐NHS	24.40/0.67	50/1.6	2.0 × 10^−3^/2.5 × 10^−3^	1.0/1.2
P(Arg‐OH‐AAm_50_‐stat‐NAM_50_)/Cy5‐NHS	27.71/0.67	50/1.4	1.8 × 10^−3^/2.2 × 10^−3^	1.0/1.2
P(Arg‐OH‐AAm_75_‐stat‐NAM_25_)/Cy5‐NHS	31.02/0.67	50/1.3	1.6 × 10^−3^/1.9 × 10^−3^	1.0/1.2
P(Arg‐OH‐AAm)/Cy5‐NHS	34.24/0.67	50/1.2	1.5 × 10^−3^/1.8 × 10^−3^	1.0/1.2

### Determination of the Hydrophilic/Hydrophobic Ratio

The polarity ratio of the copolymers was conducted as described previously in De Breuck et al.,^[^ ^39^ ^]^ albeit with minor modifications. The fluorescently labeled copolymers were dissolved in 30% glycerol in D‐PBS (0.1 mg mL^−1^, control). 100 µl of each sample (= reference) was transferred into a well of a 96‐well microtiter plate, and the fluorescence intensity was measured with a multi‐label plate reader (Tecan GENios Pro, Männedorf, Switzerland; Ex/Em = 612nm/670 nm; Software: XFLUOR4GENIOSPRO, Version: V 4.53). Then, the remaining sample solutions were mixed in a 1:1 ratio with chloroform and vortexed for 1 min. After an incubation time of 15 min at room temperature, the samples formed an aqueous and an organic layer. To determine the fluorescence signal after phase separation, 100 µl of the aqueous and organic layer, respectively, of each sample were transferred into wells of a 96‐welll microtiter plate, and the fluorescence signal was quantified spectrophotometrically. The fluorescent intensity of each copolymer (compared to the reference) in each layer was calculated as follows (Equation 4):

(4) $$\begin{array}{ccc} & & fluorescence_{normalized}\;\left(\%\right)\\ & & \;=\frac{fluorescence_{aqueous\;or\;organic\;layer\;}-fluorescence_{blank}}{fluorescent\;intensity_{reference}-fluorescence_{blank}}x\;100 \end{array}$$

### Dynamic Light Scattering (DLS) and Electrophoretic Light Scattering (ELS) Measurements

To characterize the formed copolymers, all kinds of copolymer/protein mixtures were analyzed by DLS and ELS on a Zetasizer Nano‐ZS (Malvern Panalytical Ltd, Worcestershire, UK; He–Ne laser, λ = 633 nm) with the following settings: The automatic mode was set to a measurement temperature of 37.0 ± 0.1 °C (DLS) or 25.0 ± 0.1 °C (ELS), respectively, to obtain either the number‐weighted size distribution of the copolymers for the estimation of the polymer/protein diameter or the zeta potential (with capillary cuvettes: DTS1070, Malvern Panalytical Ltd) by the Smoluchowski approximation of the Henry equation.

The following conditions were measured using DLS: Solvent comparison measurements in Dulbecco's phosphate buffered saline (D‐PBS), cell culture medium, and 30% glycerol in D‐PBS (0.5 mg mL^−1^ copolymer); For protein interaction experiments (solvent: 30% glycerol in D‐PBS), dissolved copolymers (1 mg mL^−1^) were mixed in a 1:1 ratio with bovine serum albumin (BSA; 1 mg mL^−1^) or lysozyme (1 mg mL^−1^), respectively, and analyzed at different time points (t = 0, t = 24). All samples for protein fouling experiments were incubated at 37 °C during shaking (100 rpm).

To determine the size distribution and pI of the different copolymers, DLS and ELS measurements were performed at different pH values (pH 3, 4, 5, 6, 7, 8, 9, 10; pH adjustment with 10 mM HCl or NaOH) for all copolymers.

### Cell Cultivation

Cell culture experiments were conducted with human breast cancer cells (MDA‐MB‐231) and mouse fibroblasts (L929) grown in DMEM/F12, containing 10% (v/v) FBS and 1% Pen/Strep in the case of MDA‐MB‐231 cells or incubated in DMEM low glucose containing 10% FBS and 1% Pen/Strep in the case of L929 cells. Cell proliferation was ensured by incubation at 37 °C and 5% CO_2_ in a humidified atmosphere.

### 3‐(4,5‐Dimethylthiazol‐2‐yl)‐2,5‐diphenyltetrazoliumbromid (MTT) Assay

The biocompatibility of the zwitterionic copolymers was tested, as described previously in Braksch et al.^[^ ^28^ ^]^ For this purpose, the five types of Arg‐OH‐AAm‐copolymers were analyzed, for eleven different copolymer concentrations (0.005, 0.01, 0.02, 0.04, 0.08, 0.16, 0.31, 0.63, 1.25, 2.50, and 5.00 mg mL^−1^), for L929 cells, and for 24h. For this purpose, cells were seeded at a density of 10 000 cells/well (96‐well plate) and incubated overnight. Then, the medium was replaced with medium supplemented with the different copolymer amounts and incubated for 24 h. After the incubation time, the medium was discarded, the cells were washed three times with D‐PBS (100 µl per well) and then incubated (3 h, 37 °C) with MTT‐containing medium (1 mg mL^−1^). Next, 100 µL DMSO per well was added, and the cells were incubated in the dark for 15 min during shaking. The absorbance of the incubated solution was measured at an excitation wavelength of 580 nm (Tecan GENios Pro), and the values obtained were finally normalized to the control each (cells without copolymers; Equation 5).

(5) $$cell\;viability\;\left(\%\right)=\frac{Absorbance_{sample\;}-Absorbance_{blank}}{Absorbance_{negative\;control}-Absorbance_{blank}}x\;100$$

### Cellular Interaction Experiments

The interaction of fluorescently labeled copolymers was investigated by flow cytometer experiments. For this purpose, 6‐well microtiter well plates were cultivated with two different cell lines, either MDA‐MB‐231 or L929 cells, at a cell density of 400 000 cells per well. After an incubation time of 24 h at 37 °C, the fluorescently labeled copolymers (0.04 mg mL^−1^) were added to the cells and incubated with them for 4, 6, or 24 h at 37 °C (control: cells without copolymers).

After completion of the incubation time, a washing step (1 mL D‐PBS) followed, and the cells were detached from the surface with 0.25% (w/v) trypsin/ EDTA (200 µl per well). The enzyme was inactivated by adding medium to the cells (500 µL). After 3 min, the cells were centrifuged at 200 x g for 5 min to obtain the cell pellet. The supernatant was discarded, and the cell pellet was resuspended in 500 µl D‐PBS. Afterward, the flow cytometer device (BD CytoFlex S, BD Biosciences, San Jose, CA) with an integrated bandpass filter Ex/Em = 690nm/650 nm and a solid‐state diode laser (50 mW; λ_ex_ = 488 nm) was used to measure the fluorescence signal of the cell population (10 000 events/ sample at slow detection rates of 15 µl min^−1^). Flow Jo software (BD Biosciences, v10.10.0) was used as an analysis tool.

The authors would like to acknowledge Prof Andreas Greiner, Prof Thomas Scheibel, Prof Ruth Freitag, and Prof Georg Papastavrou for providing access to their laboratories and equipment. The authors thank Dr. Valérie Jérôme for support with flow cytometry analysis, Lys Sprenger for her help with CD measurements, as well as Jan A. Kurki for the synthesis of AEAM‐Cl. This project was generously supported by the German Research Foundation (DFG; project number 535904448). M.N.L. further acknowledges financial support from the “Fonds der Chemischen Industrie im Verband der Chemischen Industrie.” M.H. gratefully acknowledges funding by the Emmy‐Noether‐Program of the German Research Foundation (Deutsche Forschungsgemeinschaft, DFG; HA 7725/2‐1, project number 445804074) as well as support by the University Research Focus “Sustainable Materials Design ” of the University of Potsdam. T.M.L. acknowledges funding by the Deutsche Forschungsgemeinschaft (DFG, German Research Foundation) – Project‐ID 391977956 – SFB 1357.

Open access funding enabled and organized by Projekt DEAL.

## Conflict of Interest

The authors declare no conflict of interest.

## Supporting information



Supporting Information

## Data Availability

The data that support the findings of this study are available from the corresponding author upon reasonable request.

## References

[1] S. Mitragotri , J. Lahann , Adv. Mater. 2012, 24, 3717. 22807037 10.1002/adma.201202080

[2] a) A. Komin , L. Russell , K. Hristova , P. Searson , Adv. Drug Del. Rev. 2017, 110, 52; 10.1016/j.addr.2016.06.002 27313077

[3] L. Yin , C. Yuvienco , J. K. Montclare , Biomaterials 2017, 134, 91. 28458031 10.1016/j.biomaterials.2017.04.036 PMC5513498

[4] a) A. O. Elzoghby , W. M. Samy , N. A. Elgindy , J. Controlled Release 2012, 161, 38; 10.1016/j.jconrel.2012.04.036 22564368

[5] F. Kratz , J. Controlled Release 2008, 132, 171. 10.1016/j.jconrel.2008.05.010 18582981

[6] D. Olsen , C. Yang , M. Bodo , R. Chang , S. Leigh , J. Baez , D. Carmichael , M. Perälä , E.‐R. Hämäläinen , M. Jarvinen , Adv. Drug Del. Rev. 2003, 55, 1547. 10.1016/j.addr.2003.08.008 14623401

[7] C. Gonzalez‐Obeso , E. J. Hartzell , R. A. Scheel , D. L. Kaplan , Adv. Drug Del. Rev. 2023, 192, 114622. 10.1016/j.addr.2022.114622 PMC9812964 36414094

[8] E. Krayukhina , K. Tsumoto , S. Uchiyama , K. Fukui , J. Pharm. Sci. 2015, 104, 527. 25256796 10.1002/jps.24184 PMC4359023

[9] H. J. Hsu , J. Bugno , S. r. Lee , S. Hong , Wiley Interdiscip. Rev.:Nanomed. Nanobiotechnol. 2017, 9, 1409. 10.1002/wnan.1409 27126551

[10] X. Hou , T. Zaks , R. Langer , Y. Dong , Nat. Rev. Mater. 2021, 6, 1078. 34394960 10.1038/s41578-021-00358-0 PMC8353930

[11] A. W. Du , M. H. Stenzel , Biomacromolecules 2014, 15, 1097. 24661025 10.1021/bm500169p

[12] Y. Xu , T. Fourniols , Y. Labrak , V. Préat , A. Beloqui , A. des Rieux , ACS Nano 2022, 16, 7168. 35446546 10.1021/acsnano.2c02347

[13] L. Collins , A. L. Parker , J. D. Gehman , L. Eckley , M. A. Perugini , F. Separovic , J. W. Fabre , ACS Nano 2010, 4, 2856. 20408581 10.1021/nn901414q

[14] A. N. Singh , R. D. Thakre , J. C. More , P. K. Sharma , Y. Agrawal , Polym.‐Plast. Technol. Eng. 2015, 54, 1077.

[15] a) M. N. Leiske , C. Kuenneth , J. De Breuck , B. G. De Geest , R. Hoogenboom , Macromol. Chem. Phys. 2023, 224, 2300200;

[16] F. Zhao , Y. Zhao , Y. Liu , X. Chang , C. Chen , Y. Zhao , Small 2011, 7, 1322. 21520409 10.1002/smll.201100001

[17] a) S. Fujii , S. Takano , K. Nakazawa , K. Sakurai , Biomacromolecules 2022, 23, 2846; 35486537 10.1021/acs.biomac.2c00216

[18] E. L. Lieu , T. Nguyen , S. Rhyne , J. Kim , Exp. Mol. Med. 2020, 52, 15. 31980738 10.1038/s12276-020-0375-3 PMC7000687

[19] S. He , S. Liu , J. Controlled Release 2024, 365, 919. 10.1016/j.jconrel.2023.12.017 38103789

[20] W. Lv , Y. Wang , H. Fu , Z. Liang , B. Huang , R. Jiang , J. Wu , Y. Zhao , Acta Biomater. 2024, 181, 19. 38729548 10.1016/j.actbio.2024.05.006

[21] D. Montizaan , K. Yang , C. Reker‐Smit , A. Salvati , Nanomed. Nanotechnol. Biol. Med. 2020, 30, 102300. 10.1016/j.nano.2020.102300 32931929

[22] P. P. Mapfumo , L. S. Reichel , K. Leer , J. Egger , A. Dzierza , K. Peneva , D. Fischer , A. Traeger , ACS Macro Lett. 2024, 13, 1000. 39052525 10.1021/acsmacrolett.4c00321 PMC11340021

[23] a) N. Corrigan , K. Jung , G. Moad , C. J. Hawker , K. Matyjaszewski , C. Boyer , Prog. Polym. Sci. 2020, 111, 101311;

[24] a) Y. Lee , C. Boyer , M. S. Kwon , Chem. Soc. Rev. 2023, 52, 3035; 37040256 10.1039/d1cs00069a

[25] M. Hartlieb , Macromol. Rapid Commun. 2022, 43, 2100514. 10.1002/marc.202100514 34750911

[26] A.‐C. Lehnen , J. Gurke , A. M. Bapolisi , M. Reifarth , M. Bekir , M. Hartlieb , Chem. Sci. 2023, 14, 593. 36741515 10.1039/d2sc05197d PMC9847670

[27] J. Martin , M. Michaelis , S. Petrović , A. C. Lehnen , Y. Müllers , P. Wendler , H. M. Möller , M. Hartlieb , U. Glebe , Macromol. Biosci. 2025, 25, 2400316. 39360589 10.1002/mabi.202400316 PMC11727822

[28] C. P. Braksch , A. C. Lehnen , A. M. Bapolisi , J. Gurke , J. De Breuck , M. N. Leiske , M. Hartlieb , J. Polym. Sci. 2024, 62, 132.

[29] a) M. N. Leiske , K. Kempe , Macromol. Rapid Commun. 2022, 43, 2100615; 10.1002/marc.202100615 34761461

[30] Y. Y. Khine , M. Callari , H. Lu , M. H. Stenzel , Macromol. Chem. Phys. 2016, 217, 2302.

[31] a) M. Gorman , Y. H. Chim , A. Hart , M. O. Riehle , A. J. Urquhart , J. Biomed. Mater. Res., Part A 2014, 102, 1809; 10.1002/jbm.a.34853 23784937

[32] W. A. N. El‐Din , I. O. A. Fattah , Tissue Cell 2024, 91, 102572. 39326233 10.1016/j.tice.2024.102572

[33] Y. Satoh , H. Kotani , Y. Iida , T. Taniura , Y. Notsu , M. Harada , Cancer Sci. 2020, 111, 2248. 32426941 10.1111/cas.14490 PMC7484823

[34] H. H. Jo , C.‐Y. Lin , E. V. Anslyn , Acc. Chem. Res. 2014, 47, 2212. 24892802 10.1021/ar500147x

[35] R. Ding , J. Ying , Y. Zhao , R. Soc. Open Sci. 2021, 8, 201963. 33959351 10.1098/rsos.201963 PMC8074886

[36] C. A. Fitch , G. Platzer , M. Okon , B. Garcia‐Moreno E , L. P. McIntosh , Protein Sci. 2015, 24, 752. 25808204 10.1002/pro.2647 PMC4420524

[37] A. Aliaga , C. Garrido , P. Leyton , J. S. Gomez‐Jeria , T. Aguayo , E. Clavijo , M. Campos‐Vallette , S. Sanchez‐Cortes , Spectrochim. Acta, Part A 2010, 76, 458. 10.1016/j.saa.2010.01.007 20471905

[38] S. Hayakawa , S. Nakai , J. Food Sci. 1985, 50, 486.

[39] J. De Breuck , M. Streiber , M. Ringleb , D. Schröder , N. Herzog , U. S. Schubert , S. Zechel , A. Traeger , M. N. Leiske , ACS Polym. Au 2024, 4, 222. 38882030 10.1021/acspolymersau.3c00048 PMC11177303

[40] J. Jasinski , M. V. Wilde , M. Voelkl , V. Jérôme , T. Fröhlich , R. Freitag , T. Scheibel , ACS Appl. Mater. Interfaces 2022, 14, 47277. 36194482 10.1021/acsami.2c13987

[41] K. Tokarczyk , K. Kubiak‐Ossowska , B. Jachimska , P. A. Mulheran , J. Phys. Chem. B 2018, 122, 3744. 29536734 10.1021/acs.jpcb.7b12484

[42] A. Salis , M. Boström , L. Medda , F. Cugia , B. Barse , D. F. Parsons , B. W. Ninham , M. Monduzzi , Langmuir 2011, 27, 11597. 21834579 10.1021/la2024605

[43] D. E. Kuehner , J. Engmann , F. Fergg , M. Wernick , H. W. Blanch , J. M. Prausnitz , J. Phys. Chem. B 1999, 103, 1368.

[44] S. Tomita , H. Yoshikawa , K. Shiraki , Biopolymers 2011, 95, 695. 21509744 10.1002/bip.21637

[45] V. Vagenende , M. G. Yap , B. L. Trout , Biochemistry 2009, 48, 11084. 19817484 10.1021/bi900649t

[46] X. Chen , B. Bhandari , P. Zhou , Food Chem. 2019, 278, 780. 30583443 10.1016/j.foodchem.2018.11.117

[47] M. Bernard , E. Jubeli , J. Bakar , L. Tortolano , J. Saunier , N. Yagoubi , J. Biomed. Mater. Res., Part A 2017, 105, 3333. 10.1002/jbm.a.36199 28875577

[48] J. A. Finbloom , F. Sousa , M. M. Stevens , T. A. Desai , Adv. Drug Del. Rev. 2020, 167, 89. 10.1016/j.addr.2020.06.007 PMC10822675 32535139

[49] P. Akarsu , S. Reinicke , A. C. Lehnen , M. Bekir , A. Böker , M. Hartlieb , M. Reifarth , Small 2023, 19, 2301761. 10.1002/smll.202301761 37381652

[50] S. C. Larnaudie , J. C. Brendel , K. A. Jolliffe , S. Perrier , J. Polym. Sci., Part A: Polym. Chem. 2016, 54, 1003.

