# Supplementary material for: Bio‐Inspired, Zwitterionic Copolymers with Amphiphilic Character
Source: Macromol Rapid Commun. 2025 Mar 27;47(14):2401099. doi: 10.1002/marc.202401099 (PMC13384794; doi:10.1002/marc.202401099)
Supplement: Supplementary file 1 — Supporting Information [file MARC-47-2401099-s001.pdf]

Supporting Information

Bio-inspired, zwitterionic copolymers with amphiphilic character

Theresa M. Lutz[1], *Cevin P. Braksch*[2], *Jonas De Breuck*[1], *Matthias Hartlieb*[2,4], *Meike N. Leiske*[1,3,*]

Dr T. M. Lutz, J. De Breuck, Prof Dr M. N. Leiske
Macromolecular Chemistry, University of Bayreuth,
Universitätsstraße 30, 95447 Bayreuth, Germany
E-mail: meike.leiske@uni-bayreuth.de

C. P. Braksch, Dr M. Hartlieb
Institute of Chemistry, University of Potsdam
Karl-Liebknecht-Str. 24-25, 14476 Potsdam, Germany

Prof Dr M. N. Leiske
Bavarian Polymer Institute
Universitätsstraße 30, 95447 Bayreuth, Germany

Dr M. Hartlieb
Fraunhofer Institue for Applied Polymer Research (IAP),
Geiselbergstraße 69, 14476 Potsdam, Germany

1. Copolymers – a broad range of properties based on the composition.

Selected copolymers were synthesized (see library; **Figure S1A-E**) allowing for an initial investigation of (self-)interaction and thus, agglomeration, intermolecular binding properties with proteins and cells, and accessible functional groups for post-modification. Several copolymer variants are formed in which the Arg-OH-AAm amount per molecule differs resulting in a broad variety of physiochemical properties (*e.g.*, hydrophobicity, see **Table S1 and S2**).

Table S1: Selected monomers for the synthesis of copolymers with different Arg-OH-AAm content. The table contains the amounts, material quantities, and equivalents of the individual monomers used for XPI-RAFT polymerization.

Polymer	Arg-OH-AAm			NAM			AEAAm-HCl		
	m (mg)	n (mmol)	equiv.	m (mg)	n (mmol)	equiv.	m (mg)	n (µmol)	equiv.
P(NAM)	-	-	-	489.2	3.5	99.0	5	35	1
P(Arg-OH-AAm$_{25}$-*stat*-NAM$_{75}$)	195.7	0.9	24.5	368.1	2.6	74.5	5	35	1
P(Arg-OH-AAm$_{50}$-*stat*-NAM$_{50}$)	395.4	1.7	49.5	244.6	1.7	49.5	5	35	1
P(Arg-OH-AAm$_{75}$-*stat*-NAM$_{25}$)	595.2	2.6	74.5	121.1	0.9	24.5	5	35	1
P(Arg-OH-AAm)	790.9	3.5	99.0	-	-	-	5	35	1

Table S2: Amounts, material quantities as well as equivalents of the different chain transfer agents are listed in the following table. Moreover, the solvent volumes for each copolymer synthesis are shown in detail.

Polymer	PABTC			Xan			dioxane/H_2O (1:1)
	m (mg)	n (µmol)	equiv.	m (mg)	n (µmol)	equiv.	V (mL)
P(NAM)	7.5	31.5	90	0.7	3.5	10	3.1
P(Arg-OH-AAm$_{25}$-*stat*-NAM$_{75}$)	7.5	31.5	90	0.7	3.5	10	3.0
P(Arg-OH-AAm$_{50}$-*stat*-NAM$_{50}$)	7.5	31.5	90	0.7	3.5	10	2.9
P(Arg-OH-AAm$_{75}$-*stat*-NAM$_{25}$)	7.5	31.5	90	0.7	3.5	10	2.8
P(Arg-OH-AAm)	7.5	31.5	90	0.7	3.5	10	2.7

Moreover, circular dichroism (CD) measurements reveal negative signals in a range of 200 to 240 nm, thus we assume that mainly L-Arg were retained during the synthesis (**Figure S1F**).

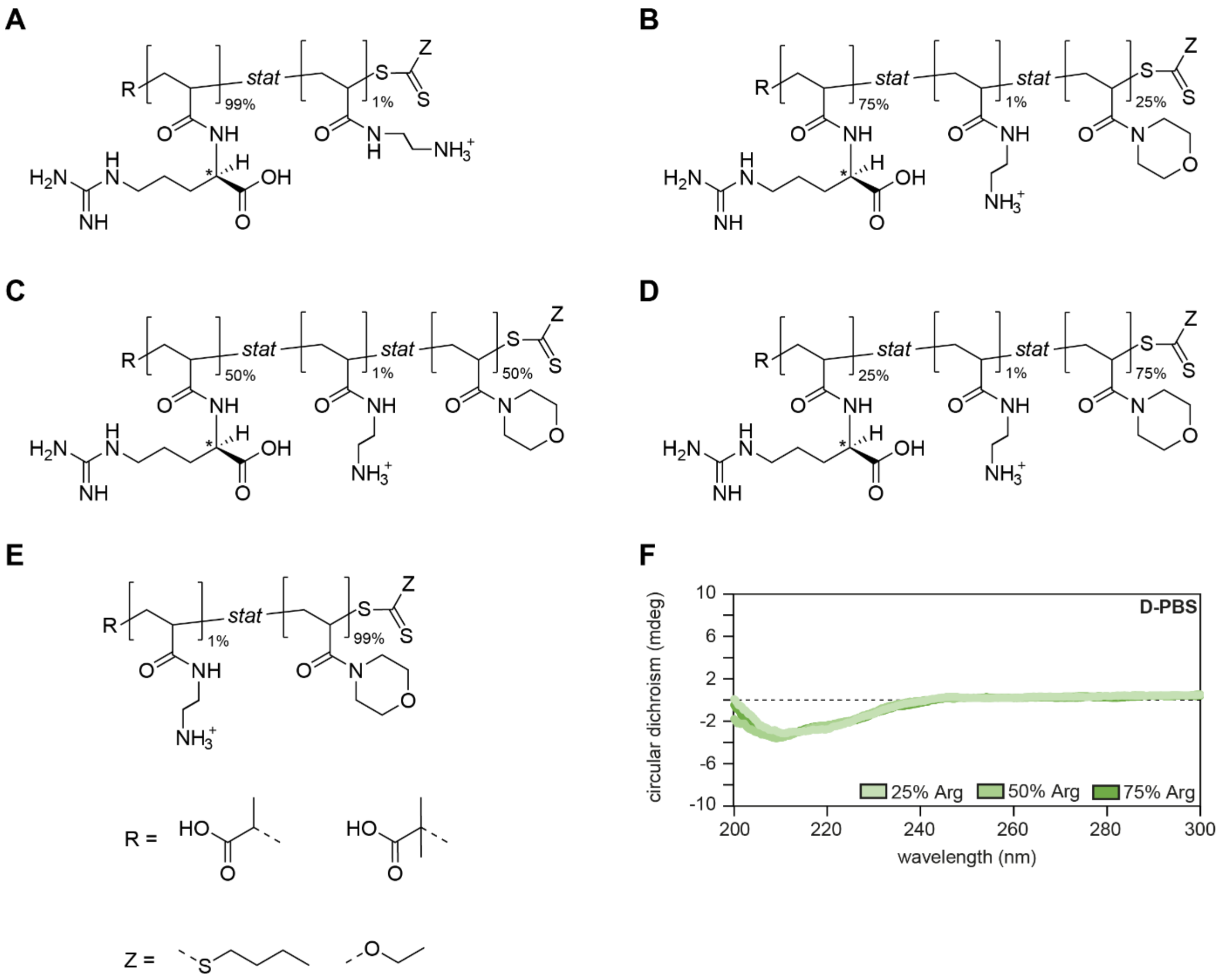

Figure S1: ***Overview of the copolymer library characterized in more detail in this study, as well as, enantiomer investigation.*** The copolymers' main core is established by Arg-OH-AAm motifs of up to ~100% (**A**), 75% (**B**), 50% (**C**), 25% (**D**), and 0% (**E**). Stereochemical measurements obtained by circular dichroism revealed L-Arg moieties.

The quality of copolymer synthesis over time (**Figure S2**), and the analysis of monomer conversion into copolymers was investigated by NMR (see main paper; **Figure 2A**).

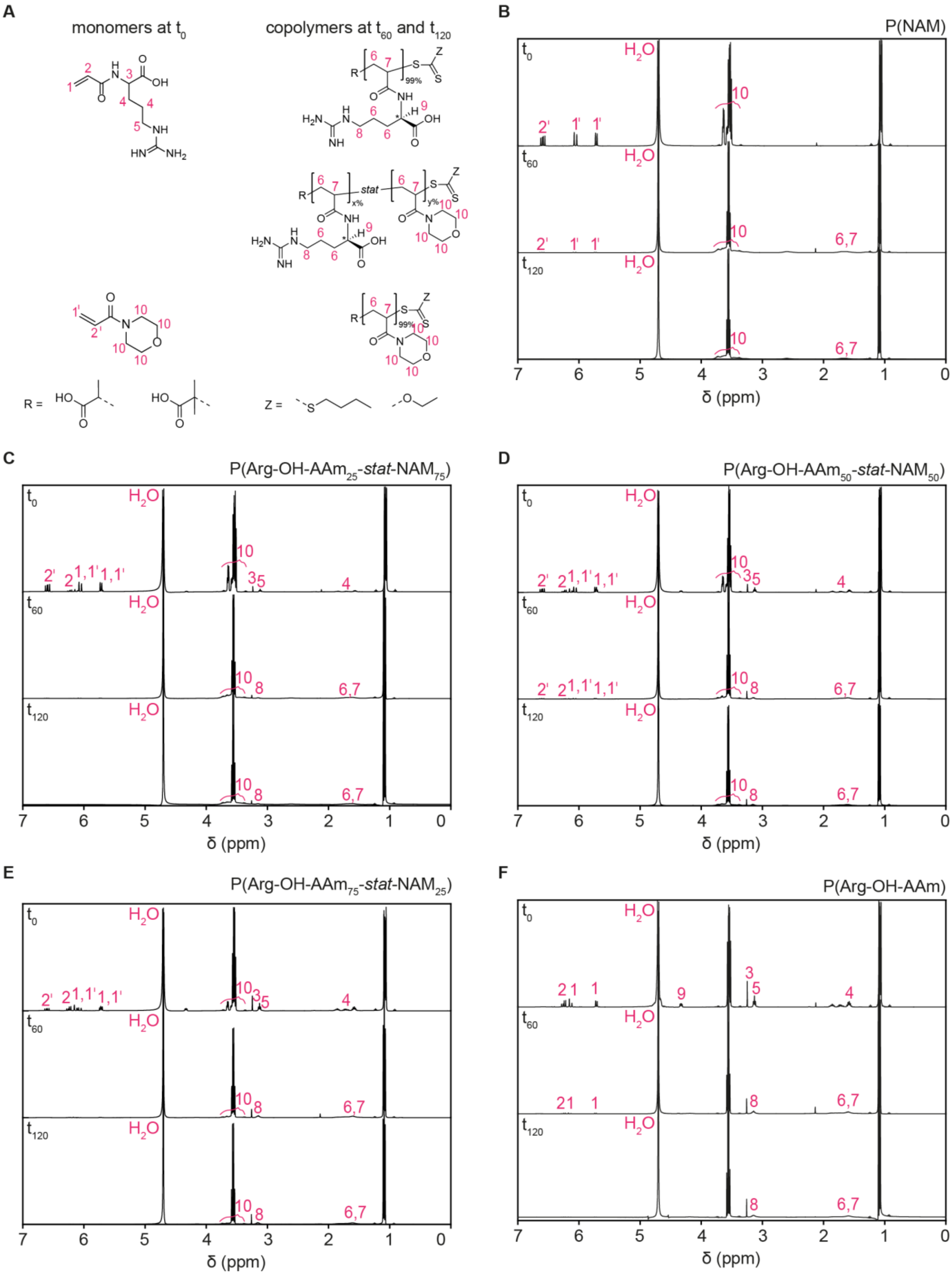

Figure S2: ***Representative ^{1}H NMR data show the kinetic of monomer conversion from 0 min to 120 min.*** (**A**) Chemical structures of monomers and copolymers investigated with NMR. ^{1}H NMR spectra for 0% (**B**), 25% (**C**), 50% (**D**), 75% (**E**), and 100% (**F**) Arg-OH-AAm modified polymers at different time points (400 MHz; D_2O).

The copolymers showed a high monomer conversion, which is associated with a well-controlled synthesis strategy. The theoretical mean molar distribution and theoretical dispersity was listed in **Table S3**. Moreover, further parameters such as the apparent mean molecular weight ($M_{n, app}$) and the dispersity values of each copolymer were determined by SEC measurements (**Table S3**).

Table S3: The table contains the polymer properties such as monomer conversion, X_{theo}, $M_{n,theo}$, $M_{n, app}$, and Đ obtained by ^{1}H NMR (400 MHz; light grey), SEC measurements in 80:20 Water/ACN mixture supplemented with 0.1 M NaCl, and 0.1 V% TFA (Poly(2-vinylpyridine) standard, PSS calibration kit; medium grey), and SEC measurements in H_2O containing 0.1M NaCl and 0.3 % formic acid (Poly(ethylene glycol) (PEG) calibration, PSS calibration kit; dark grey).

Polymer	conv. Arg-OH-AAm (%)	conv. NAM (%)	$X_{n,theo}$ Arg-OH-AAm	X_{theo} NAM	$M_{n,theo}$ (kDa)	$M_{n,app}$ (kDa)	Đ	$M_{n,app}$ (kDa)	Đ
P(NAM)	-	96	-	192	43.8	0.27	1.32	5.3	1.26
P(Arg-OH-AAm$_{25}$-*stat*-NAM$_{75}$)	100	97	50	146	32.0	0.8	1.47	12.6	1.49
P(Arg-OH-AAm$_{50}$-*stat*-NAM$_{50}$)	99	98	99	98	36.4	12.1	1.99	18.2	1.69
P(Arg-OH-AAm$_{75}$-*stat*-NAM$_{25}$)	98	98	147	49	40.5	15.3	1.80	27.3	2.62
P(Arg-OH-AAm)	98	-	196	-	44.7	19.4	4.28	31.5	3.72

SEC measurements in aqueous solvent showed the molar mass distribution of the individual, synthesized (co)polymers **(Figure S3)**. We obtained monomodal/narrow peaks for hydrophilic polymers (0%-50% Arg-OH-AAm), whereas the hydrophobic ones exhibited broader peaks and thus higher molecular masses (75%-100% Arg-OH-AAm).

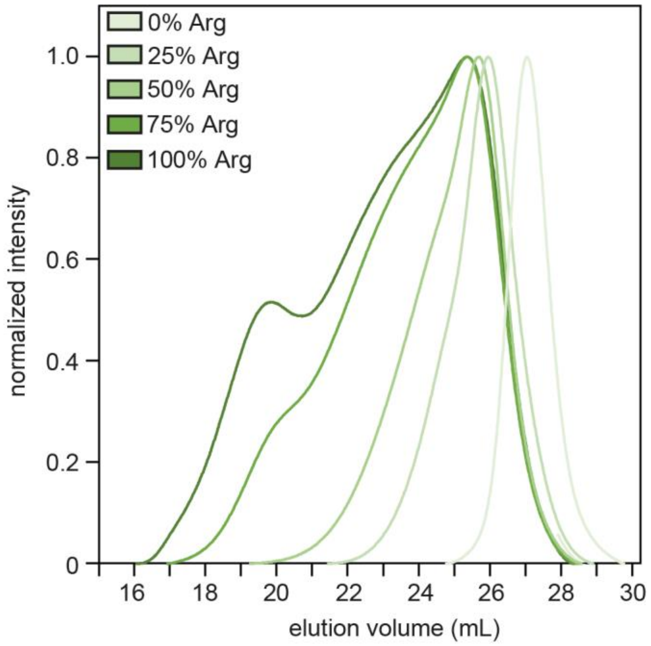

Figure S3: ***Characterization of the synthesized copolymers with SEC.*** (**A**) SEC measurements in H_2O containing 0.1M NaCl and 0.3 % formic acid (Poly(ethylene glycol) (PEG) calibration) show the molecular weight distribution of the tested copolymers.

2. Solvents having an important impact on protein/copolymer solubility and interaction.

To characterize the physicochemical properties and binding interactions of the formed copolymer species, Dynamic and Electrophoretic Light Scattering (DLS & ELS) measurements were determined in different solvents and conditions. The mean number diameter of those copolymer copolymers in D-PBS has shown values of ~5-8 nm (Arg-OH-AAm content of 0%, 25%, and 50%) or ~1,000 nm (75% Arg-OH-AAm motifs), respectively (**Figure S4A**). This more contracted structure of the copolymers was less pronounced at different pH values. Here, the mean number diameter of the copolymers was ~10 to ~100 nm (Arg-OH-AAm content: 0%, 25%, and 50%) or ~100 nm to ~1,000 nm (Arg-OH-AAm content: 75% and 100%) at pH 5 to pH 10 (**Figure S4B**). The intramolecular interaction (compact structure formation of individual copolymers) of copolymers with low Arg-OH-AAm content (up to 50%) and the intermolecular aggregation of copolymers with high Arg-OH-AAm amounts (75% and 100%) is comparably low at pH 3 and 4. This is made possible by the presence of ions leading to the formation of agglomerates[1], *i.e.*, when the copolymers were exposed to sodium hydroxide (NaOH; NaOH

concentration increased with rising pH values), the mean number diameter increased systematically (**Figure S4B**).

Glycerol, however, further enhanced the globular protein structure (BSA and lysozyme: ~0.8 nm mean number diameter), whereas the buffer solution only was without effects on the contracted state (BSA: ~6 nm; lysozyme: ~4 nm; **Figure S4C**). The copolymeric mean number diameter (~4-6 nm) during BSA or lysozyme interaction in D-PBS (**Figure S4D and E**) hardly differs from the incubation of corresponding, individual copolymers in D-PBS (**Figure S4A**). Only the mean number diameter of copolymers with 75% Arg-OH-AAm content was altered after 24 h (from ~1,000 nm to ~4-6 nm). Thus, a more compact variant was formed, which was similar to the values obtained for the other three copolymers (**Figure S4D and E**). Those number diameter changes (t_0; 75% Arg-OH-AAm content) were driven by unspecific binding interactions such as entanglement between proteins and polymers[2]. The copolymers seem to resist interaction with cell culture medium components since we obtained similar log mean number diameters compared to DLS measurements with FBS in cell culture medium (**Figure S4F**). Only the subset of copolymers with 50% Arg-OH-AAm content seemed to inhibit agglomeration formation of FBS after 24h. However, we expect that there was no negative influence on cell experiments.

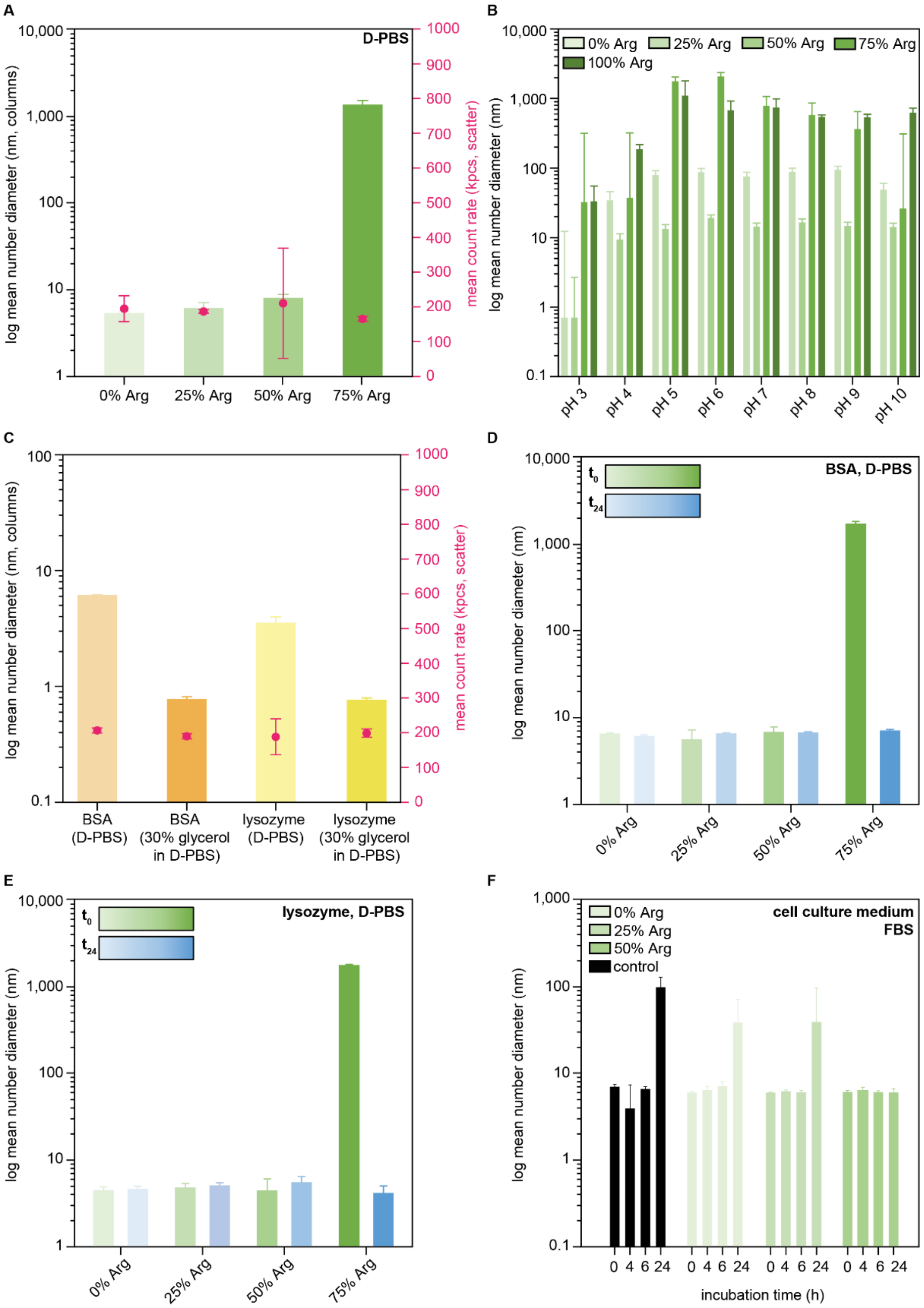

Figure S4: ***Mean number diameter changes of copolymers and/or proteins in different solvents.*** (**A, B**) To test the solubility of the copolymers, DLS measurements are assessed in D-PBS and in 30% glycerol in D-PBS at different pH values (from 3 to 10). (**C**) The solvent can influence the mean number diameter of BSA and lysozyme. (**D, E**) Qualitatively similar results are observed for copolymer/protein interactions at t_0 and t_{24} during incubation at 37°C. (**F**) DLS measurements to assess the mean number diameter of different copolymers in cell culture medium. (**A-F**) The error bars represent the standard error of the mean from three replicates.

For an overview the size and number distribution (at t_0 and t_{24}) of individual proteins (**Figure S7**) or a subset of copolymers incubated with BSA and lysozyme (**Figure S5 and S6**), respectively, in glycerol/D-PBS were plotted and compared. Except for the two copolymers with higher Arg-OH-AAm concentrations (75 %), the graphs showed peaks which perfectly matched the individual results for BSA/lysozyme and copolymers indicating no binding interaction between the molecules and thus, prevention of protein fouling. In contrast, exposing the copolymers with 75% Arg-OH-AAm motifs to proteins revealed indistinguishable peaks of the two molecule classes (agglomerate formation).

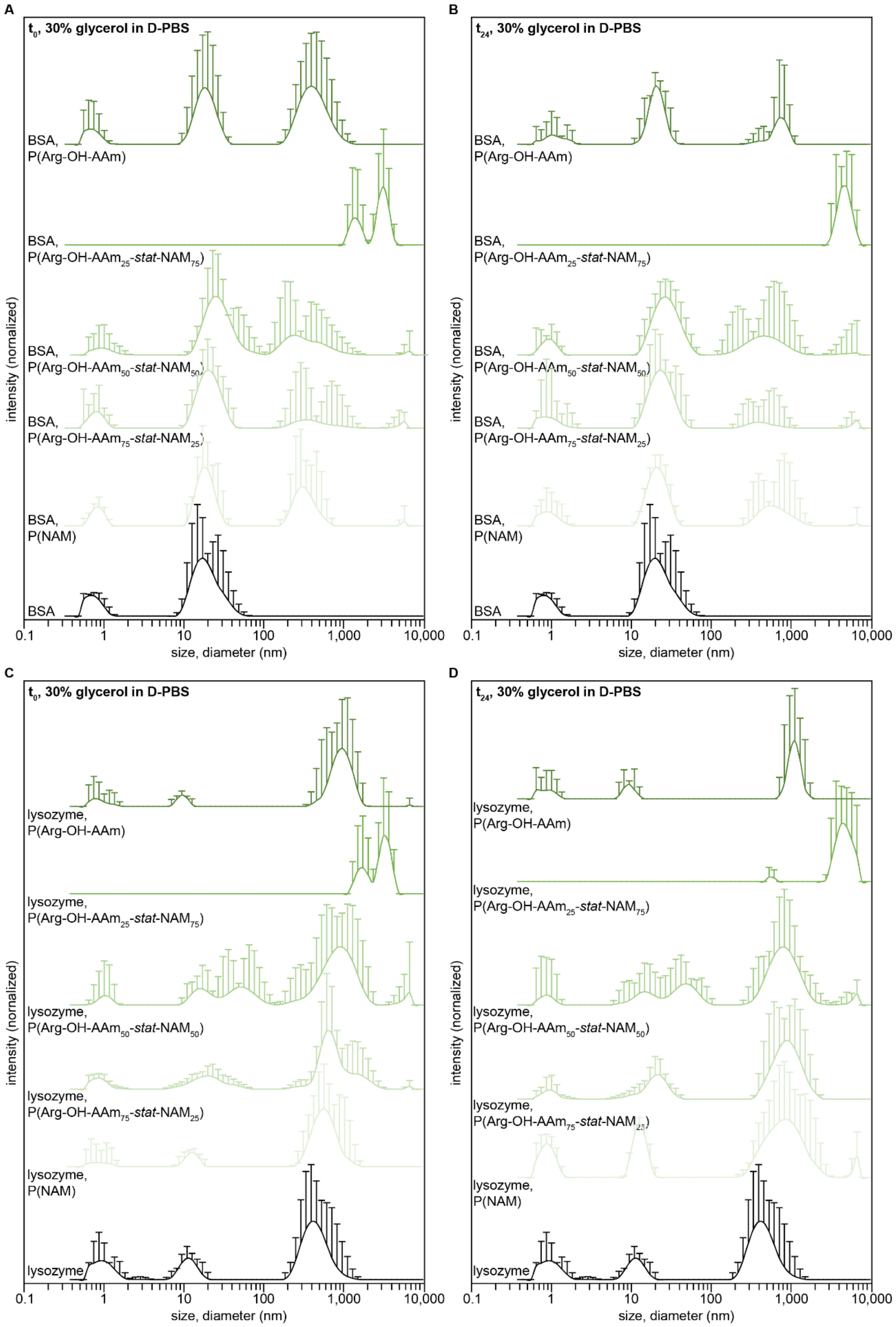

Figure S5: ***Size alterations of copolymer/protein mixtures in glycerol/D-PBS over time.*** DLS measurements show the size distribution of copolymers with 0%, 25%, 50%, 75%, and 100% Arg-OH-AAm content interacting with BSA or lysozyme for 0h (**A,C**) and 24h (**B,D**) at 37°C. The error bars show the standard error of the mean as obtained from three independent samples.

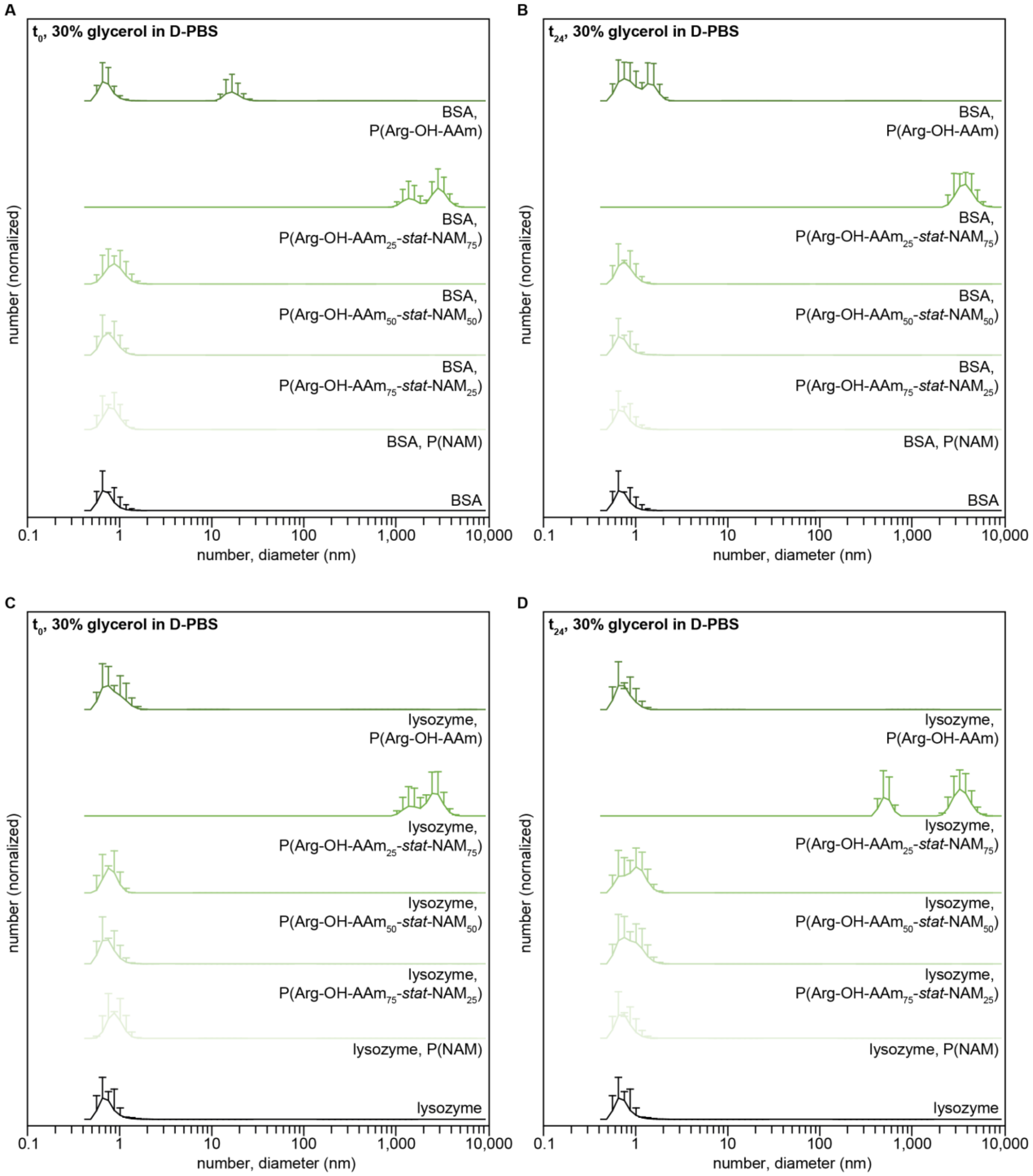

Figure S6: ***Qualitative assessment of copolymer interaction for different time points.*** The number diameter of copolymers with different Arg-OH-AAm motif amounts (0%, 25%, 50%, 75%, 100%) used in this study was determined by DLS measurements as a function of the BSA/lysozyme interaction for 0h (**A,C**) and 24 h (**B,D**). The error bars indicate the standard error of the mean obtained for an ensemble of at least three independent measurements.

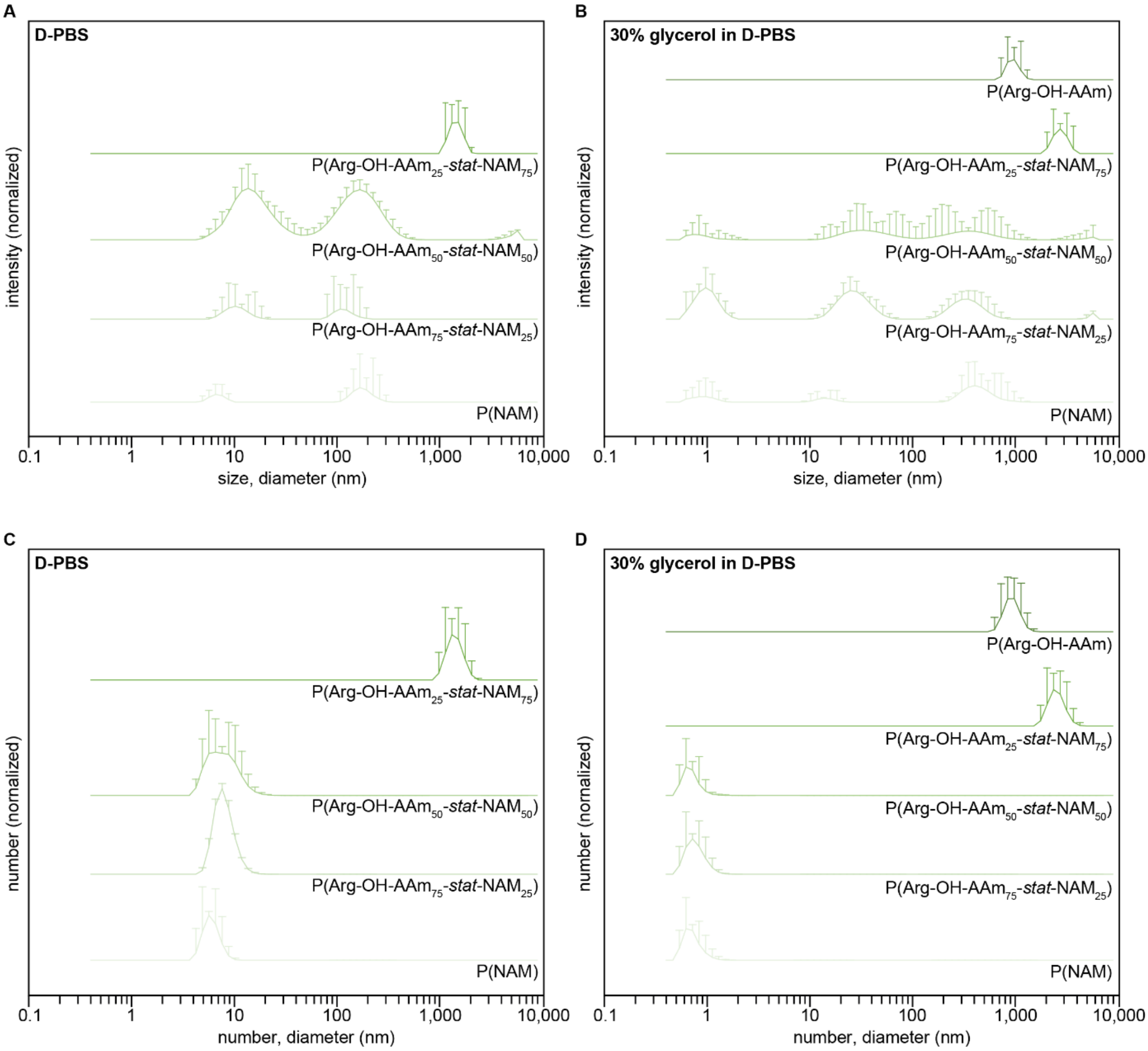

Figure S7: ***DLS measurements show alterations in the size and number diameter of copolymers with different*** **Arg-OH-AAm** ***content.*** The size (**A,B**) and number diameter (**C,D**) distribution of copolymers used in this study were analyzed by DLS in D-PBS (**A,C**) and 30% glycerol in D-PBS (**B,D**). The error bars show the standard error of the mean as obtained from three independent samples.

3. Generating fluorescently labeled copolymers by carbodiimide coupling.

To determine, for example, the copolymer hydrophilicity and interaction with cells, covalent labeling of the different copolymer variants with Cy5 (**Scheme S1**) allowed for analysis of the copolymer distribution in aqueous/organic solvents and the illustration/quantification of fluorescence signals in cellular structures.

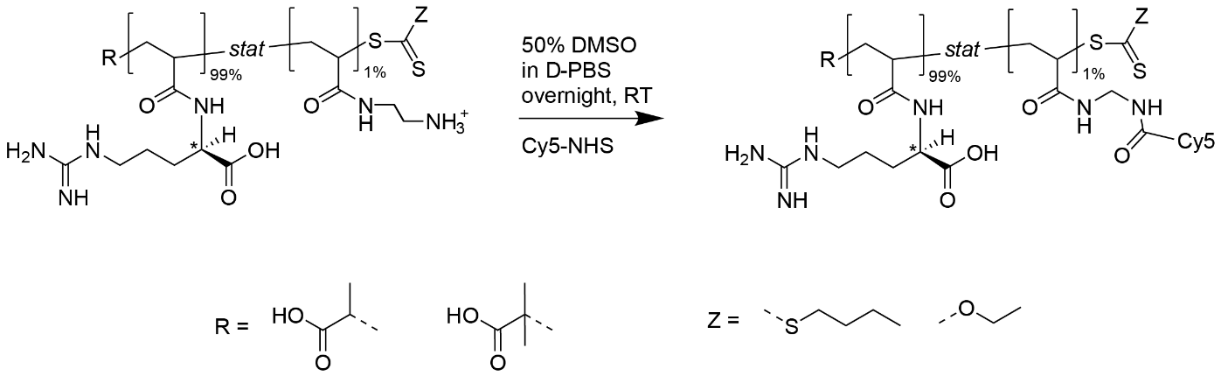

Scheme S1: ***Schematic representation of fluorescent labeling of copolymers.*** Shown is the modification process for copolymers with ~100% Arg-OH-AAm content. The amino groups of the copolymer are functionalized with Cy5-NHS in DMSO/D-PBS to form a suitable copolymer for further analysis.

The success of copolymer labeling with Cy5 was determined with two methods: The first option was based on SEC (**Table S4 and Figure S8A**) and the second strategy was an optical verification process (**Figure S8B**). By using the polystyrene (PS) standard, the mean molecular weight (M_n) of the water-soluble polymers was estimated. Accordingly, the copolymers without Arg-OH-AAm showed a $M_{n,app}$ = 4,090 g mol^{-1}, the copolymers with 25% and 50% Arg-OH-AAm motifs a $M_{n,app}$ = 9,500 g mol^{-1} and $M_{n,app}$ = 10,980 g mol^{-1}, respectively.

Table S4: The table shows the copolymer properties such as $M_{n,\ app}$, and Đ measured with SEC in 80:20 Water/ACN mixture supplemented with 0.1 M NaCl, and 0.1 V% TFA (P2VP-cal.).

Polymer	$M_{n,app}$ (kDa)	Đ
Cy5-P(NAM)	4.09	1.28
Cy5- P(Arg-OH-AAm$_{25}$-*stat*-NAM$_{75}$)	9.53	1.33
Cy5- P(Arg-OH-AAm$_{50}$-*stat*-NAM$_{50}$)	10.98	1.34

The blue color intensity of the fluorescently labeled copolymers in solution, as analyzed by the naked eye, indicated, on the one hand, that the binding of Cy5 was successfully and, on the other hand, that the copolymers (without Arg-OH-AAm and low Arg-OH-AAm content up to 50%) are soluble in chloroform and glycerol/D-PBS. However, the polymers with a high Arg-OH-AAm content (75% and 100%) were unable to dissolve in either organic or aqueous liquids.

These results were very important in terms of determining polarity ratios (assay conditions based on solubility in chloroform and/or glycerol/D-PBS) and for the use of Cy5-modified copolymers for cell interaction studies (aqueous conditions). Therefore, detection measurements with Cy5-modified polymers (SEC, flow cytometer, and hydrophobicity measurements) were limited to three copolymers (Arg-OH-AAm content: 0%, 25%, and 50%).

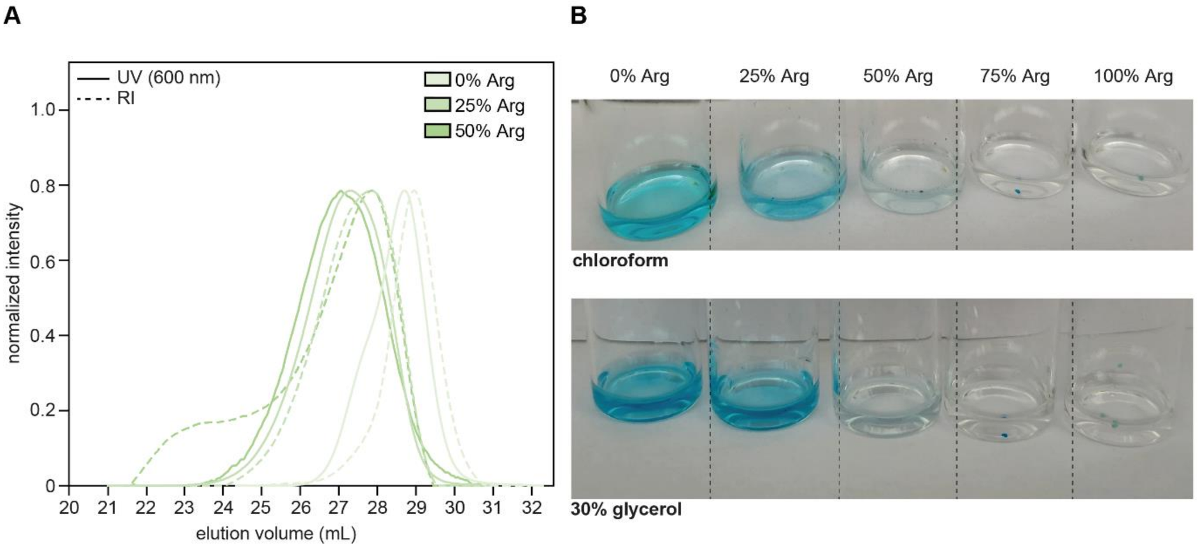

Figure S8: ***Assessing the mean molecular weight distribution and solubility of Cy5-labeled copolymers.*** (**A**) SEC measurements in 80:20 Water/ACN mixture containing 0.1 M NaCl, and 0.1 V% TFA (P2VP-cal.) show monomodal peaks of the tested copolymers. (**B**) Copolymers without Arg-OH-AAm and low Arg-OH-AAm contents (≤ 50%) were dissolved in aqueous/organic solvents.

4. Copolymer interaction and internalization studies with eukaryotic cells.

To assess the biocompatibility of the formed copolymers, solutions of different copolymer concentrations were prepared in cell culture medium and incubated with L929 cells at 37°C for 24h. After conducting an MTT assay, the obtained viability values were calculated (**Figure S9**). Only high polymer concentrations (≥ 0.63 mg mL^{-1}) with 0% and 50% Arg-OH-AAm content show an inhibitory effect on cellular biocompatibility.

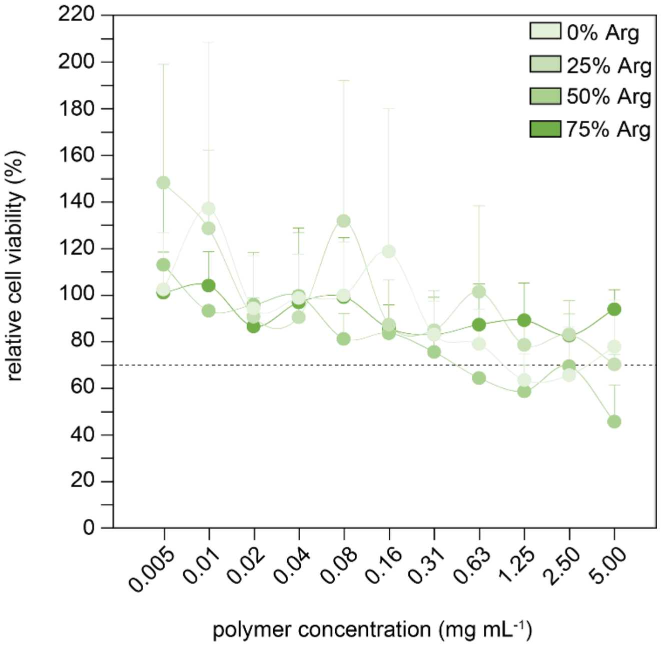

Figure S9: ***Effect of copolymers on the viability of L929 cells.*** The biocompatibility was analyzed with various polymer concentrations using MTT assay. The error bars denote the standard error of the mean as obtained from independent analytical triplicates.

In particular, the interaction of Cy5-labeled polymers (0%-50% Arg-OH-AAm content) with mouse fibroblasts (= non-cancer cells) and human epithelial breast cancer cells was investigated. For this purpose, flow cytometer measurements served as detection method. To achieve this, a gating set up was required: First, cell debris was removed from the measurements (**Figure S10A**) and then, false positive fluorescent signals were excluded by gating for singlet cells (**Figure S10B**). Subsequently, we quantified the fluorescence of cells interacting with Cy5-labeled copolymers (**Figure S10C**), which was discussed in more detail in the main paper.

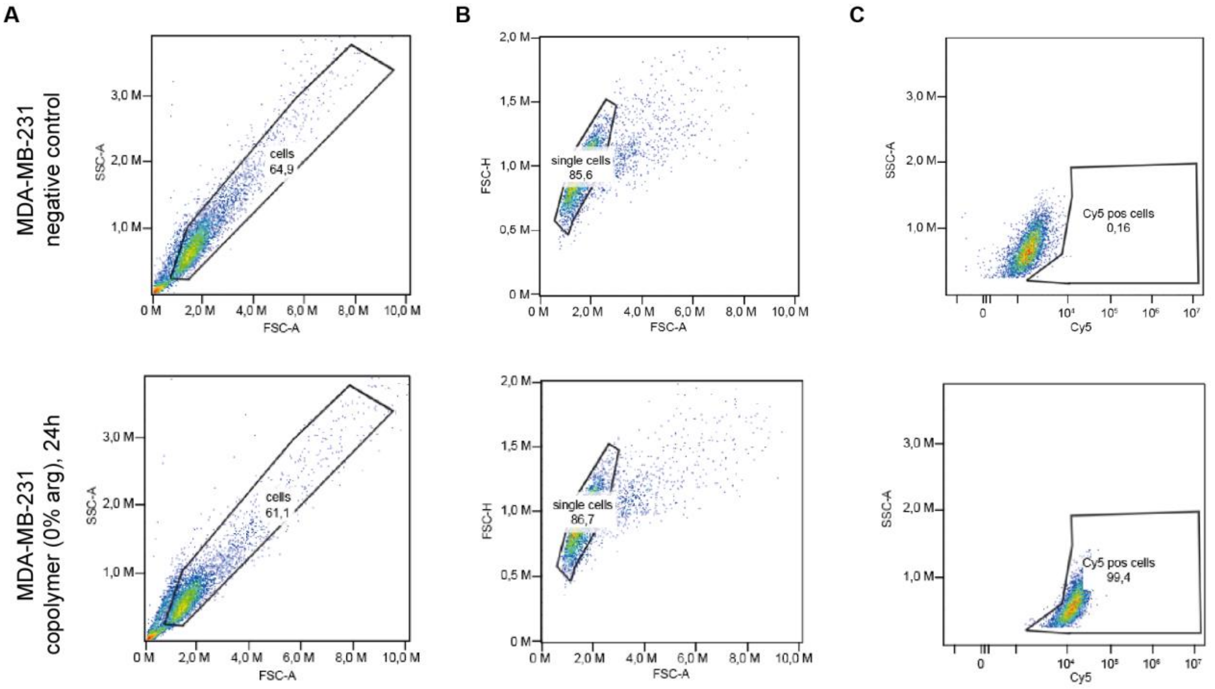

Figure S10: ***Gating strategy to assess copolymer-cell interactions using flow cytometry.*** (**A**) First, viable cells were **detected** and gated by forward scattering (FSC) versus side scattering (SSC). (**B**) In the next step, singlet cells were addressed by FSC vs. FSC plotting. (**C**) To analyze the Cy5 positive events, the negative control (cells without polymers) was excluded form gating ensuring detection of fluorescent cells only.

References

[1] A. S. Lee, A. P. Gast, V. Bütün, S. P. Armes, *Macromolecules* **1999,** *32* (13), 4302-4310.
[2] A. Huang, J. M. Paloni, A. Wang, A. C. Obermeyer, H. V. Sureka, H. Yao, B. D. Olsen, *Biomacromolecules* **2019,** *20* (10), 3713-3723.
